# Evaluating variation in human gut microbiota profiles due to DNA extraction method and inter-subject differences

**DOI:** 10.3389/fmicb.2015.00130

**Published:** 2015-02-18

**Authors:** Brett Wagner Mackenzie, David W. Waite, Michael W. Taylor

**Affiliations:** Centre for Microbial Innovation & Centre for Brain Research, School of Biological Sciences, University of AucklandAuckland, New Zealand

**Keywords:** human gut microbiota, nucleic acids extraction, beta diversity, PERMANOVA

## Abstract

The human gut contains dense and diverse microbial communities which have profound influences on human health. Gaining meaningful insights into these communities requires provision of high quality microbial nucleic acids from human fecal samples, as well as an understanding of the sources of variation and their impacts on the experimental model. We present here a systematic analysis of commonly used microbial DNA extraction methods, and identify significant sources of variation. Five extraction methods (Human Microbiome Project protocol, MoBio PowerSoil DNA Isolation Kit, QIAamp DNA Stool Mini Kit, ZR Fecal DNA MiniPrep, phenol:chloroform-based DNA isolation) were evaluated based on the following criteria: DNA yield, quality and integrity, and microbial community structure based on Illumina amplicon sequencing of the V4 region of bacterial and archaeal 16S rRNA genes. Our results indicate that the largest portion of variation within the model was attributed to differences between subjects (biological variation), with a smaller proportion of variation associated with DNA extraction method (technical variation) and intra-subject variation. A comprehensive understanding of the potential impact of technical variation on the human gut microbiota will help limit preventable bias, enabling more accurate diversity estimates.

## Introduction

The human gut harbors the most substantial microbial communities within our bodies, with these communities exhibiting considerable inter- and intra-personal variability (Eckburg et al., 2005; Ley et al., 2006; Qin et al., 2010). Several diseases and disorders have been linked to dysbiosis (imbalance) in these gut communities, and recent studies have sought to identify changes to microbial community structure and function during health and disease (Bäckhed et al., 2004; Cantarel et al., 2012; Claesson et al., 2012; Qin et al., 2012; Hsiao et al., 2013; Gevers et al., 2014). Gut microbial community composition varies less within an individual than among different individuals, suggesting a strong environmental component (Turnbaugh et al., 2006; Qin et al., 2010; Caporaso et al., 2011; The Human Microbiome Project Consortium, 2012; Schloissnig et al., 2013).

Nucleic acids extraction from stool samples is the first step in describing microbial diversity using culture-independent methods. Differences in lysis efficiency, heterogeneous distribution of microbes across a sample, and adherence of microbes to the stool matrix can result in preferential lysis of certain cell types and misrepresentation of microbial community diversity (Li et al., 2003; Ariefdjohan et al., 2010; Maukonen et al, 2012). DNA extraction methods utilize different lysis procedures, such as mechanical (bead beating), chemical, enzymatic, and heat, and various method comparison studies have reported contradictory results regarding the most effective lysing procedure (Carrigg et al., 2007; Dridi et al., 2009; Maukonen et al, 2012; Yuan et al., 2012; Claassen et al., 2013). Methodological, or technical, variation such as that due to different DNA extraction techniques, sequencing platforms, and/or sequence processing pipelines can influence descriptions of ecological diversity and observed biological variation, including inter-subject variation.

Although many studies have compared DNA extraction protocols for the gut microbiota (McOrist et al., 2002; Li et al., 2003; Yu and Morrison, 2004; Nechvatal et al., 2008; Lauber et al., 2010; Bahl et al., 2012; Maukonen et al, 2012; Yuan et al., 2012; Claassen et al., 2013; Henderson et al., 2013; Peng et al., 2013; Kennedy et al., 2014), a consensus as to the most efficient extraction method, which is most representative of gut microbial community diversity from stool samples, has not yet been reached. It is crucial to continually assess nucleic acids extraction methods, identifying those that are the most efficient, accurate and reproducible with the overall aim to standardize methods across gut microbiology studies, limiting preventable bias due to technical variation. Although a recent meta-analysis of stool samples showed strong clustering by experimental protocol, namely choice of PCR primer (Lozupone et al., 2013), the individual impacts from the sources of variation, including inter- and intra-subject variation and laboratory technique, have not been previously quantified.

This study examined multiple stool samples from three age- and sex-matched individuals using several DNA extraction methods to identify and quantify the degree to which choice of DNA extraction method (technical variation) impacted upon inter-subject variation (biological variation). In addition, data from an existing study of the human fecal microbiota (Yatsunenko et al., 2012) were included in order to further assess our predictions about inter-subject variability.

## Materials and methods

### Subject selection and stool sample collection

Subjects for this study were chosen from a database of self-registered volunteers. Ethics approval for this project was granted by the Northern B Health and Disability Ethics Committee (Ref. 12/NTB/59). Three female subjects in their mid-20s (Subjects 1, 2, and 3), with no dietary restrictions, gastrointestinal disturbances or recent antibiotic usage (>6 months) were chosen for this study. All subjects were located within Auckland, New Zealand and consented to supply three entire stool samples at 48 h intervals into sterile polypropylene containers. Stool samples were immediately stored on ice until processing of the samples in the lab. Pre-processing of samples into smaller sub-samples occurred within 12 h of collection and these were stored at -80°C until microbial DNA was extracted. Samples for the Human Microbiome Project (HMP) extraction method were processed first, before homogenization, according to the HMP Manual of Procedures 2010 Version 11.0 (McInnes and Cutting, 2010). After sub-samples were processed according to the HMP protocol, the remaining stool sample was homogenized via stirring with a sterile metal spatula and sample sizes were standardized to 200 mg, enabling better comparisons between the DNA extraction methods.

### DNA extraction

DNA was extracted in triplicate from each stool sample using five commonly used microbial DNA extraction methods, namely the Human Microbiome Project extraction method (HMP), MoBio PowerSoil® DNA Isolation Kit (M), QIAamp® DNA Stool Mini Kit (Q), ZR Fecal DNA MiniPrep™ (Z), and one non-kit phenol:chloroform-based DNA isolation protocol (P) (Zoetendal et al., 2006) (Table 1). All bead-beating steps were performed in the Qiagen TissueLyser II at a frequency of 30 Hz for 2 min and centrifugation steps were carried out in an Eppendorf 5415D centrifuge.

**Table 1:** **Comparison of the five microbial DNA extraction methods used in this study**.

**Extraction method**	**Kit abbreviation**	**Recommended fecal starting amount (mg)**	**Lysis Type**	**Elution volume (μL)**	**DNA Isolation**
Human Microbiome Project Extraction Method	HMP	1 mL supernatant	Heat, Mechanical	100	Spin column
MoBio PowerSoil^®^ DNA Isolation Kit	M	250	Mechanical	100	Spin column
Qiagen QIAamp^®^ DNA Stool Mini Kit	Q	180–220	Heat, Chemical, Enzymatic	200	Spin column
Zymo ZR Fecal DNA MiniPrep™	Z	150	Mechanical	100	Spin column
Phenol: chloroform-based DNA isolation	P	200	Mechanical	100	Phase separation

*A total of 135 samples were analyzed from 5 extraction methods, comprising 3 sub-samples from each of 3 entire stool samples from 3 subjects*.

#### The human microbiome project consortium extraction method

The HMP method uses the MoBio PowerSoil® DNA Isolation Kit, but includes a pre-processing protocol, in which 1 mL of supernatant from a centrifuged mixture of 2 mL stool sample and 5 mL MoBio lysis buffer, was added to MoBio bead tubes for two 10 min heating steps at 65°C then 95°C prior to freezing at −80°C (McInnes and Cutting, 2010). The only subsequent deviation from the standard MoBio PowerSoil® DNA Isolation Kit protocol was a longer centrifugation step after the addition of Inhibitor Removal Technology® Solution C3 to precipitate a DNA pellet from any non-DNA organic and inorganic material, including humic acids, cell debris, and proteins. All other extraction steps were followed according to the manufacturer's instructions.

#### MoBio PowerSoil® DNA Isolation Kit

Samples were thawed on ice and added to the PowerBead Tubes provided in the kit using a sterile metal spatula. The MoBio PowerSoil® DNA Isolation Kit involves mechanical lysis of cells during a bead-beating step. Humic substances were then removed using patented Inhibitor Removal Technology. A salt solution was added to help the DNA bind to the silica spin column filter before the DNA was washed with an ethanol-based solution to remove residual salt, humic acids or any other contaminants. Last, a sterile elution buffer (10 mM Tris) released the DNA from the spin column filter, yielding DNA that is ready for downstream applications.

#### QIAamp® DNA Stool Mini Kit

After samples were thawed on ice, Buffer ASL was added to help lyse bacterial cells and samples were placed in the Tissue Lyser II for 2 min at 30 Hz. The homogenate was then incubated in a 70°C water bath for 5 min. Stool particles were pelleted, then an InhibitEX Tablet was added to the supernatant to adsorb inhibitors. Proteinase K and Buffer AL were added to the supernatant to digest proteins. The DNA was bound to a spin column filter and impurities were washed from the sample using 96–100% ethanol and proprietary Buffer AW2. The extracted microbial DNA was eluted with 200 μL Buffer AE.

#### ZR fecal DNA MiniPrep™ kit

ZR Fecal DNA MiniPrep™ is a bead-beating and spin-column filter extraction kit. Similar to other bead-beating kits, a lysis solution was added to the sample in the ZR BashingBead™ Lysis Tube to help lyse bacterial cells. Fecal DNA Binding Buffer and centrifugation in the spin column tube then bound the extracted DNA to the spin filter. The bound DNA was washed, and an extra elution step resulted in twice-filtered DNA.

#### Phenol:chloroform-based DNA isolation

This protocol for DNA extraction by phase separation was adapted from Zoetendal et al. (2006). Samples were thawed on ice and added to 2 mL tubes containing 0.3 g of 0.1 mm diameter silica zirconia beads. Buffer-saturated phenol was added to the tube containing the stool sample and beads, and homogenized in the Tissue Lyser II for 2 min at 30 Hz. The homogenized sample was briefly cooled on ice before adding chloroform and isoamyl alcohol (24:1). A 3 M sodium acetate salt solution was used to bind the extracted DNA from the upper aqueous phase, while 95% ethanol washed away impurities. The resulting DNA pellet was dried on the bench top, then rehydrated in 100 μL TE Buffer.

### Evaluation of DNA yield, quality and integrity

Final yield and quality of extracted DNA were determined spectrophotometrically using the NanoDrop® ND-1000 (NanoDrop Technologies Inc., Wilmington, USA). DNA yield was also determined fluorometrically using the Broad Range (BR) kit on the Qubit® Fluorometer 1.0 (Invitrogen Co., Carlsbad, USA). Pure genomic DNA is indicated by an A260/A280 nm ratio between 1.8 and 2.0. Integrity of genomic DNA was determined by visualizing 3 μL of extracted DNA on a 1% agarose gel (w/v) containing SYBR Safe DNA Gel Stain (Invitrogen Co., Carlsbad, USA) run in 0.5X TBE buffer at 100 V for 45 min. All values were normalized to 200 mg starting material and 100 μL elution volume, to allow for accurate comparisons between methods.

### Statistical analyses of DNA yield and quality data

Statistical analyses, including coefficient of variation (CV) tests for reproducibility, Shapiro-Wilk test for normality, Kruskal-Wallis group test, and Mann-Whitney U pairwise tests with Benjamini-Hochberg (“BH”) *p*-value adjustment for multiple pairwise tests (Benjamini and Hochberg, 1995), were conducted on quantitative and qualitative data. Mean values for yield and quality were determined. All quantitative and qualitative statistical analyses were conducted in “R” version 2.15.0 (R Development Core Team, 2012).

### PCR amplification and illumina MiSeq preparation

Extracted DNA was diluted to 5 ng/μL in PCR-grade water for PCR amplification targeting the hypervariable V4 region of the bacterial and archaeal 16S rRNA genes (Caporaso et al., 2010c; Klindworth et al., 2013). PCR was performed using an Applied Biosystems® GeneAmp® PCR System 9700. Each PCR reaction contained: 2.5 μL 10X High Fidelity PCR Buffer, 1 μL 50 mM magnesium sulfate, 0.5 μL 2.5 mM dNTPs, 0.1 μL Platinum® Taq DNA Polymerase High Fidelity, 18.4 μL PCR-grade autoclaved water, and 0.5 μL 10 μM 515 F primer (5′—AAT GAT ACG GCG ACC ACC GAG ATC TAC ACT ATG GTA ATT GTG TGC CAG CMG CCG CGG TAA—3′ Caporaso et al., 2010c) composed of an Illumina adapter, a forward primer pad, a two-base linker sequence (“GT”) and the 16S rRNA gene-targeting primer (underlined). To each PCR reaction, 1 μL of each DNA template was added together with 1 μL of 5 μM unique 806R-barcoded reverse fusion primers (5′—CAA GCA GAA GAC GGC ATA CGA GAT NNNNNNNNNNNN GTG ACT GGA GTT CAG ACG TGT GCT CTT CCG ATC TGG ACT ACH VGG GTW TCT AAT—3′ Klindworth et al., 2013), composed of the Illumina adapter, a unique 12-base error-correcting Golay barcode, the reverse primer pad, a two-base linker sequence (“CC”) and the 806R primer (underlined). A positive control of *Escherichia coli* genomic DNA and a negative control of 1 μL randomly selected reverse primer with PCR-grade autoclaved water was performed for each PCR. Thermocycling conditions were: initial denaturation at 94°C for 3 min followed by 30 cycles of denaturation at 94°C for 45 s, annealing at 55°C for 45 s, and extension at 72°C for 90 s. A final extension step was performed at 72°C for 10 min.

Amplifications were completed in triplicate and 20 μL aliquots of each sample from the three PCR amplifications were pooled. After checking amplified PCR products on a 1% agarose gel (w/v) containing SYBR Safe DNA Gel Stain, pooled products were purified using the MoBio UltraClean®-htp 96 Well PCR Clean-Up Kit (MoBio Laboratories, Carlsbad, CA, USA). The manufacturer's protocol was followed and the only deviation was to increase the centrifugation time to 4 min to achieve the appropriate force in the centrifuge. All purified samples were measured fluorometrically using the High Sensitivity (HS) kit on the Qubit® Fluorometer 1.0 (Invitrogen Co., Carlsbad, USA). Impurities and contaminants were assessed for a random selection of samples using an Agilent DNA 1000 chip (Agilent Technologies, Inc., Waldbronn, Germany).

Purified PCR products were diluted in 10 mM Tris to achieve equimolar concentrations across all samples and 2 μL of each sample was combined into a sterile microcentrifuge tube. Illumina MiSeq 2 × 250 bp paired-end sequencing was performed by the Centre for Genomics, Proteomics and Metabolomics through NZ Genomics Ltd at the University of Auckland. Sequence data were uploaded to the NCBI Sequence Read Archive, project number SRP051334.

### Sequence data processing

Forward and reverse paired-end sequence reads were merged according to the fastq-join parameter (Aronesty, 2011) within the join_paired_ends.py command in QIIME (Caporaso et al., 2010b). The unique.seqs command in the mothur software identified unique sequences (Schloss et al., 2009). Abundance data were appended to unique sequences and operational taxonomic units (OTUs) were constructed using the UPARSE pipeline, based on the program usearch (Edgar, 2013). Briefly, individual OTUs were constructed by binning sequences into clusters of greater than 97% sequence similarity (-cluster_otus, -minsize 1), discarding putative chimeric OTUs in the process. A representative sequence of each OTU was further tested for chimeric artifacts using the SILVA reference database provided as part of the UPARSE pipeline (-uchime_ref). Abundance data were then reincorporated into the dataset by mapping the initial sequences against the representative OTUs (-usearch_global, -id 0.97). The resulting table was converted to biom format for analysis in QIIME using the inbuilt “convert” function of the biom software package.

Rarefaction was performed using the command alpha_rarefaction.py as part of the calculate_core_analyses.py command within QIIME 1.8 (Caporaso et al., 2010b). A total of 100 permutations were performed at 10 equal intervals between the minimum (1) and maximum (11,739) sequence depths. Alpha diversity was estimated for each DNA extraction method using the “observed species” metric within QIIME. Shannon and Simpson diversity indices measuring richness and evenness, were used to estimate diversity within each of the DNA extraction methods. Taxonomy was assigned to each sequence using uclust consensus taxonomy assigner, based on 97% sequence similarity with the Greengenes reference database version 13.5 (DeSantis et al., 2006; Caporaso et al., 2010a). A phylogenetic tree was built using FastTree 2.1.3 (Price et al., 2009) and weighted UniFrac, unweighted UniFrac, and Bray-Curtis dissimilarity matrices were generated to perform beta diversity measures on the data set (Lozupone and Knight, 2005). All three beta diversity measures returned overall similar results, and we chose to use the unweighted UniFrac metric to compare beta diversity, because of its previous successful application distinguishing microbial communities within the human microbiome (Lozupone and Knight, 2005; Wu et al., 2011; Lozupone et al., 2013). Unweighted UniFrac PCoA biplots were visualized in the EMPeror Visualization Program (Vázquez-Baeza et al., 2013).

### Statistical analyses of sequencing data

To assess which bacteria are driving the differences between Subjects and DNA extraction methods, paired, two-tailed *t*-tests, with Bonferroni adjustment for multiple pairwise comparisons, were conducted between all Subjects and DNA extraction methods for taxon-assigned OTUs.

Permutational multivariate analysis of variance (PERMANOVA) statistical analyses and pairwise tests were conducted in PERMANOVA+ in PRIMER v6 software (Anderson et al., 2008). The PERMANOVA test allows robust, unbiased analysis of multivariate data based on complex experimental designs and models (Anderson et al., 2008). PERMANOVA analyses return a *p*-value for significance and also the R^2^ value, which is indicative of the amount of variation attributed to a specific treatment within a model. PERMANOVA analysis was conducted on the unweighted UniFrac matrix, and values were obtained using type III sums of squares with 9999 permutations of residuals under a reduced model.

### Combined sequence data analysis

We combined our raw sequencing data with the raw sequencing reads from an international study conducted by Yatsunenko et al. (2012) (hereafter referred to as the Yatsunenko data) to examine predictions regarding inter-subject variation within our cohort. The same V4 region of the 16S rRNA gene was amplified in the Yatsunenko data as in our study, and our raw sequencing reads were reprocessed with the raw reads from the Yatsunenko data in accordance with the protocol used in their study. Due to the documented impact of subject age on the human gut microbiota (Lozupone et al., 2012; Yatsunenko et al., 2012), samples obtained from infants or children <3 years of age (as described in the Yatsunenko metadata) were removed from the data set. The combined data set sequences were quality filtered in QIIME, and reads <90 bp were eliminated. Representative sequences from the Yatsunenko data were aligned against the SILVA seed database and a lane mask constructed to represent the base positions covered by the first 90 nucleotides of these sequences. Our own sequences were then aligned and filtered using this lane mask, resulting in a standardization of sequence lengths. All sequences were then dereplicated in mothur, and usearch was used for closed-reference OTU picking with the Greengenes database (May 2013 release). All sample depths were normalized to 10,303 sequences per sample within the combined data sets. Taxonomy assignments and phylogenetic trees were inferred from the reference OTUs.

Two dimensional NMDS plots were generated in R (version 3.1.2) using the package vegan (version 2.2–1) (Oksanen et al., 2015) and the influence of bacterial families on the ordination was tested using the envfit function. Vectors with a statistically significant contribution to the ordination were identified following Benjamini-Hochberg (“BH”) false discovery rate correction (*p* = 0.05) and overlaid onto the ordination space. The size of taxa nodes is based upon the average abundance of the taxon in the communities. Analysis of similarity (ANOSIM) was used to identify changes in microbial community structures between countries (Clarke, 1993). ANOSIM tests between countries were performed in mothur, using 10,000 permutations to determine significance and “BH” correction applied to all *p*-values.

## Results

### Influence of DNA extraction method on yield and quality of extracted DNA

**Figure 1:**
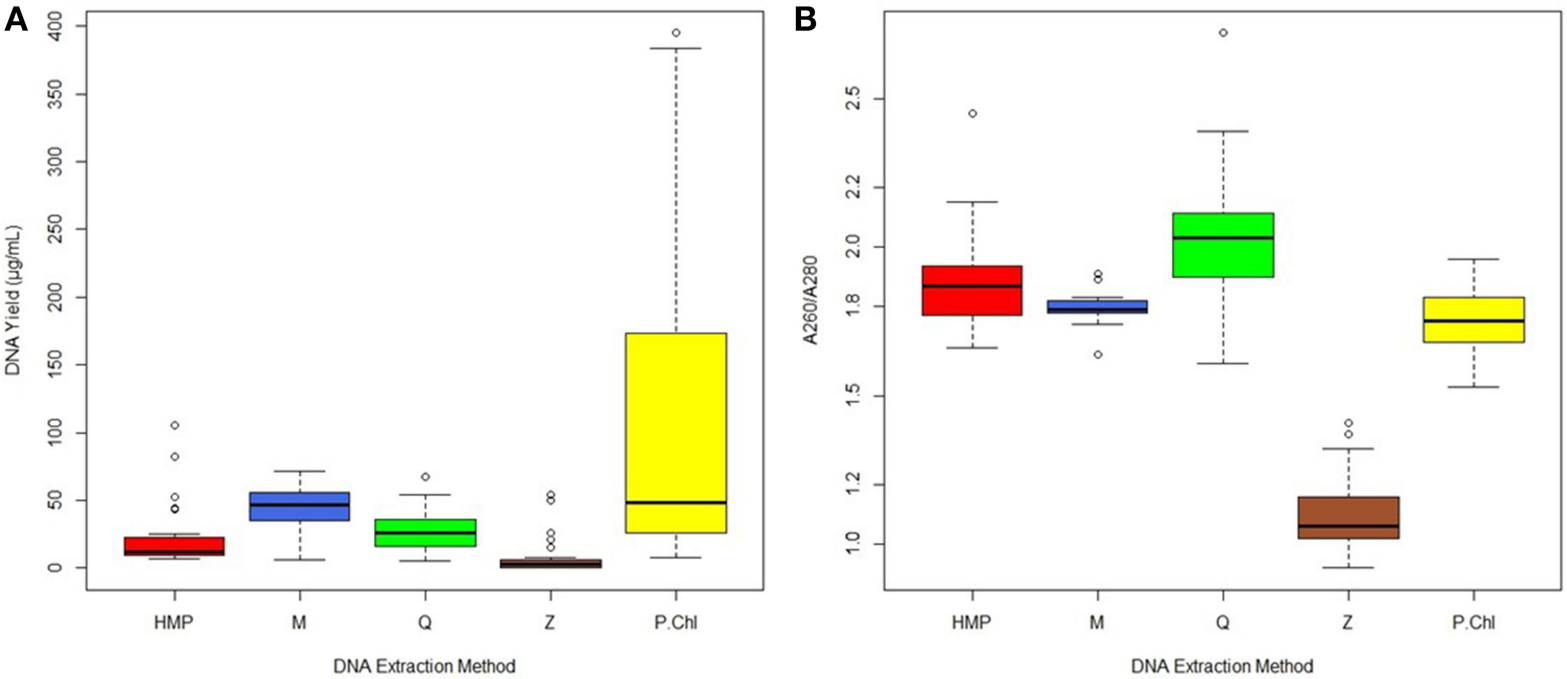
**(A)** DNA yield (μg/mL) from fecal samples (*n* = 27 samples per method), **(B)** DNA quality (A260/A280 nm ratios) from fecal samples (*n* = 27 samples per method). Median values are indicated by the solid line within each box, and the box extends to upper and lower quartile values. Outlier data points are indicated by open circles.

Mann-Whitney U pairwise tests, with “BH” *p*-value adjustment, suggest that the only pairwise comparison that did not produce significant differences for DNA yield and quality was that comparing the M and P methods (*p* = 0.12). The P method exhibited the highest median yield (59.15 μg/mL, *p* < 0.002), but was not significantly different when compared to the median yield from the MoBio PowerSoil® DNA Isolation Kit (*p* = 0.12, median yield *M* = 45.4 μg/mL). The lowest median yield (*Z* = 2.62 μg/mL, *p* < 0.001), as well as the lowest A260/A280 ratios (*Z* = 1.06, *p* < 0.001), resulted from use of method Z (Figure 1). The median value of A260/A280 ratios from M were the closest to 1.8, indicating pure, high quality DNA (HMP = 1.88, *M* = 1.79, *Q* = 2.03, *Z* = 1.06, *P* = 1.75).

Analysis of agarose gel images revealed very faint bands from Z extractions (Figure S1). These results are in agreement with the consistently low yield measurements obtained for this kit. Method P yielded a high amount of sheared DNA, visualized by the strong smear toward the bottom of the gel. Methods HMP, M, and Q showed consistently strong bands at the top of the gel, indicating high molecular weight DNA. The Human Microbiome Project gel results indicated the least amount of shearing of all the methods.

Only two kits, M and Q, proved reproducible for DNA yield with coefficient of variation (CV) values < 1 (*M* = 0.42, *Q* = 0.53). However, the median A260/A280 ratios recovered by kit Q were greater than 2.0, which is outside the range of pure DNA as indicated by the Nano Drop® ND-1000 spectrophotometer manual. All methods were reproducible for DNA quality (CV values: HMP = 0.09, *M* = 0.03, *Q* = 0.11, *Z* = 0.12, *P* = 0.07).

### Initial sequence data analysis results

A total of 135 samples were sequenced (*n* = 45 per subject, *n* = 27 per extraction method), with quality filtered (q score 30) reads resulting in 9.74 million sequencing reads with an average length of 253 bp. After removing chimeras (4.7% of the unique sequences), *de novo* OTU picking returned 5403 97%-OTUs.

### Inter-subject and DNA extraction method differences were identified as major sources of variation by permanova

**Figure 2:**
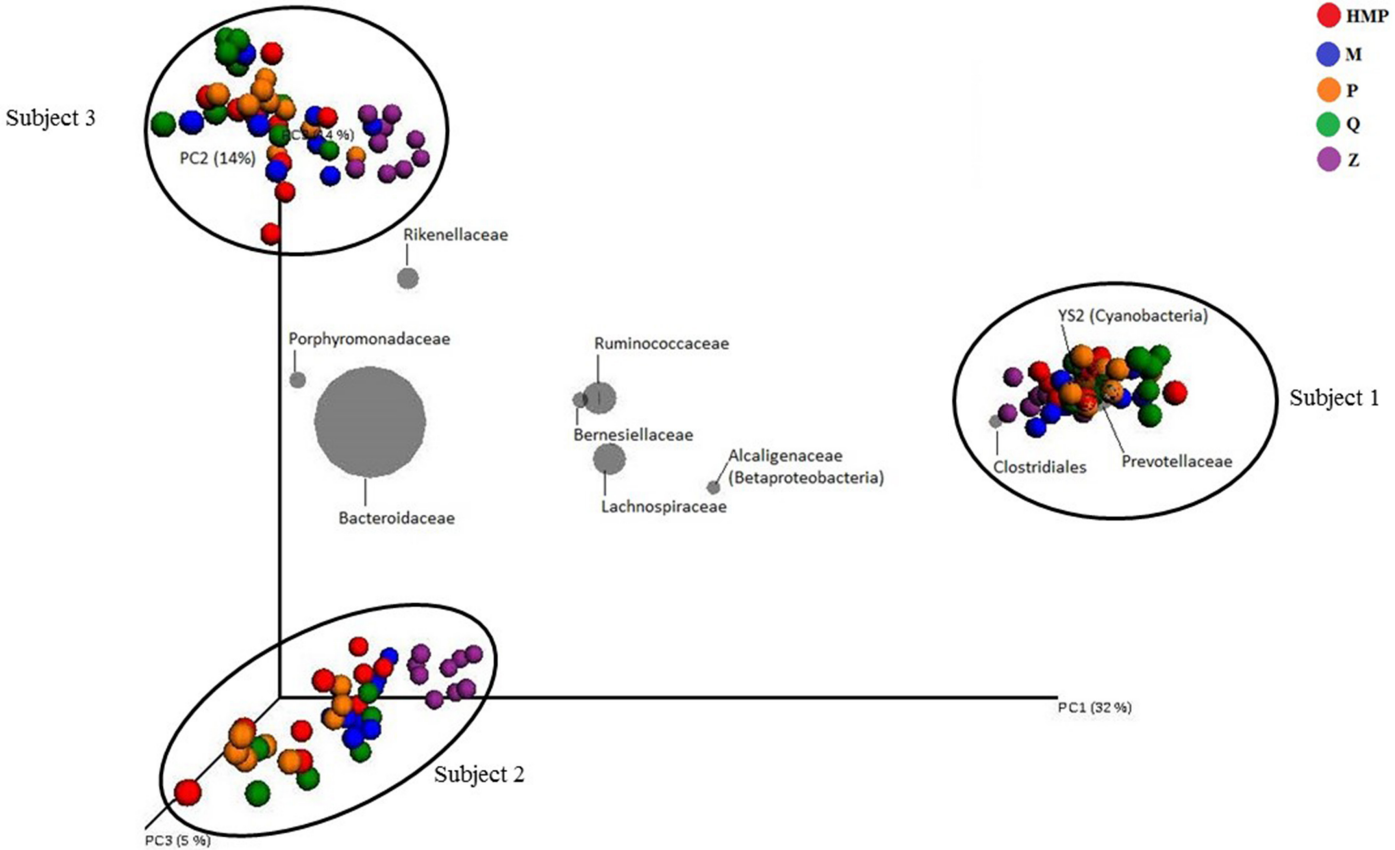
**Relative unweighted UniFrac phylogenetic distances between subjects imaged using a biplot**. Superimposed on the PCoA plot are gray spheres indicating the most abundant bacterial families. The sizes of the spheres represent the mean relative abundance of the respective taxon and the location of the spheres within the plot indicate subject-specific associations. Samples within subjects are colored by extraction method used.

PERMANOVA analysis of microbial community profiles rarefied to 11,739 sequences per sample for unweighted UniFrac revealed that the largest portion of variation could be attributed to inter-subject differences (Figure 2, Table S1). PERMANOVA analysis of weighted UniFrac and Bray-Curtis dissimilarity matrices exhibited similar overall results to those from unweighted UniFrac (results not shown). Inter-subject differences, then extraction method, and any combination thereof, all contributed significantly to observed total variation. The number of samples from individual subjects (intra-subject variation), and method combined with sample number, were not significant contributors to overall variation, suggesting that one sample per subject is sufficient for cross-sectional studies. Inter-subject differences explained 34% of the variation within the model (*R*^2^ = 0.34, *p* = 0.0001), with extraction method alone explaining 9% of variation (*R*^2^ = 0.09, *p* = 0.0006). Subject combined with extraction method explained an additional 9% of variation (*R*^2^ = 0.09, *p* = 0.0001), and subject combined with sample number explained 7% (*R*^2^ = 0.07, *p* = 0.0001). Variation between samples within each subject (intra-subject variation) did not contribute a significant proportion of variation to the model (*p* > 0.05).

Taxa biplots can be used to visualize and explore the taxonomic factors driving clustering patterns within the PCoA. In a biplot, bacterial taxa are plotted according to weighted average of taxa within all samples, where the weights of the bacterial taxa represent sequence abundance, and the location of the taxa spheres indicate which bacteria are driving clustering patterns. Taxonomic resolution at the family-level best captured the groups of bacteria driving the clustering patterns observed between subjects. The taxa biplots indicate that clustering by Subject is driven by differences in abundance of *Bacteroidaceae, Ruminococcaceae, Lachnospiraceae*, and *Prevotellaceae* (Figure 2). After primary clustering by Subject, the samples within each subject exhibit secondary clustering due to DNA extraction method. These observations are supported by the PERMANOVA results.

### Effects of DNA extraction method on microbial community profiles

DNA extraction method accounted for 9% of the variation within this experimental model. PERMANOVA pairwise tests based on unweighted UniFrac metrics revealed significant differences between HMP and Z methods (*p* = 0.044), P and Z methods (*p* = 0.021), and suggested that the largest difference in DNA extraction method was between Z and Q methods (*p* = 0.018). No significant difference between Z and M methods was detected (*p* = 0.113). No other significant differences were detected between methods for unweighted UniFrac PERMANOVA pairwise analyses.

**Figure 3:**
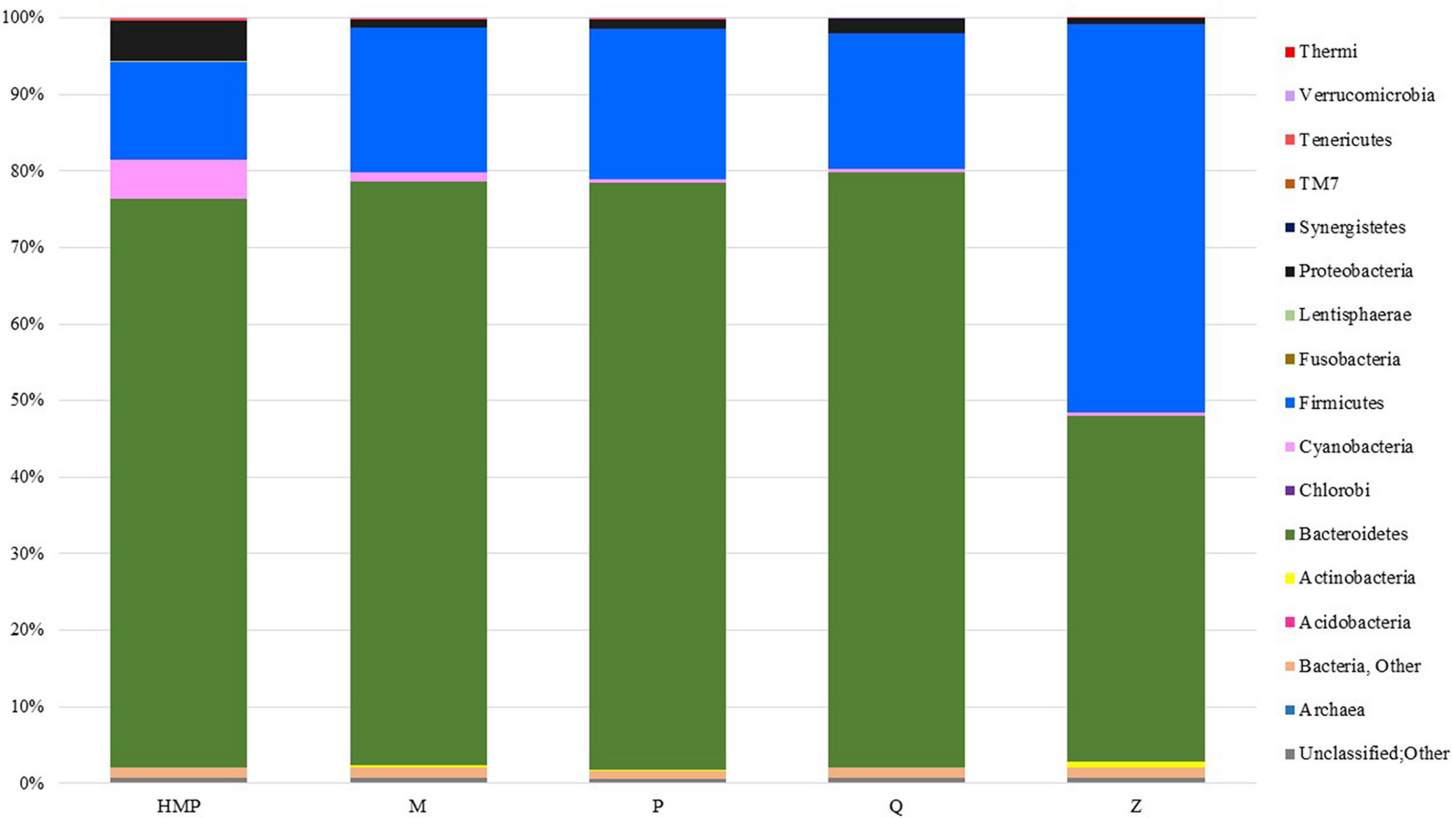
**Taxa plot summarizing the relative abundance of taxon-assigned OTUs identified for bacterial and archaeal phyla in the stool samples taken from each extraction method**. Each method represents sequencing information from 27 samples.

Members of the bacterial phyla *Bacteroidetes* and *Firmicutes* accounted for most of the taxon-assigned OTUs observed for all five extraction methods, while sequences representing *Actinobacteria, Cyanobacteria, Lentisphaerae, Proteobacteria, Tenericutes*, and *Verrucomicrobia* were also recovered using all of the extraction methods (Figure 3, Table S2). Unclassified OTUs (sequences unresolved at domain level) represented 0.58–0.75% and unclassified bacteria represented 1.0–1.3% of the total number of sequences for each of the DNA extraction methods (Table S2). The percentage of sequences represented by each phylum varied between DNA extraction methods. Method Q yielded the largest proportion of *Bacteroidetes* (77.7%), whereas Z had the lowest (45.2%), but the highest proportion of *Firmicutes* (50.8%). The HMP method yielded the lowest proportion of *Firmicutes* (12.8%); however, this method had the highest proportions of *Cyanobacteria* (5.1%) and *Proteobacteria* (5.3%) (Figure 3). Only two methods, HMP and Q, detected *Fusobacteria* (<0.001%), which is interesting since these are the only extraction methods that include a heat lysis component. Phyla that were only detected using protocol Z include *Acidobacteria, Chlorobi* and *Thermi*. HMP was the only method to detect members of the candidate phylum TM7.

Multiple pairwise comparisons between DNA extraction methods for observed OTUs richness revealed significant differences between method Z and two other methods, P (*p* = 0.02) and M (*p* = 0.04). Method Z yielded the highest number of observed OTUs, followed by the HMP method, Q, M, and P methods (Table 2). The mean Shannon diversity across all DNA extraction methods was 5.33 ± 0.51 and the mean Simpson diversity was 0.89 ± 0.06. Method Z had the highest Shannon and Simpson diversity measurements, followed by the HMP method, P, M, and Q methods (Table 2). Pairwise comparisons between the extraction methods for Shannon and Simpson indices revealed that method Z was the only kit that was significantly different from all other methods (*p* ≤ 0.002).

**Table 2:** **Mean estimates of alpha diversity metrics (mean ± S.D., *p*-values) calculated for each method from OTU tables rarefied to 11,739 sequences per sample**.

**Method**	**Chao1**	**Observed OTUs**	**Shannon diversity**	**Simpson diversity**
HMP	842 ± 64.9 (*p* = 1.0)	533 ± 44.8 (*p* = 0.32)	5.28 ± 0.41 (*p* = 0.01)	0.90 ± 0.04 (*p* = 0.02)
M	818 ± 90.1 (*p* = 0.33)	528 ± 46.0 (*p* = 0.04)	5.18 ± 0.64 (*p* = 0.01)	0.87 ± 0.09 (*p* = 0.02)
P	825 ± 61.4 (*p* = 0.24)	524 ± 38.4 (*p* = 0.02)	5.19 ± 0.52 (*p* = 0.01)	0.89 ± 0.07 (*p* = 0.01)
Q	825 ± 87.3 (*p* = 0.52)	531 ± 48.8 (*p* = 0.16)	5.13 ± 0.69 (*p* = 0.01)	0.87 ± 0.09 (*p* = 0.01)
Z	865 ± 61.0	562 ± 42.6	5.94 ± 0.30	0.94 ± 0.03

*Each method represents data from 27 samples. P-values reported in the table are only associated with pairwise comparisons with Z method. No other pairwise comparisons between methods resulted in significant p-values (p < 0.05)*.

A total of 110 OTUs significantly impacted upon the differences observed between DNA extraction methods (*p* < 0.05), of which the top 52 OTUs were members of the phylum *Firmicutes* (Table S3). Members of the *Firmicutes* family *Lachnospiraceae* were the main drivers of variation, separating Z from the other extraction methods. *Parabacteroides distasonis* sequences were found in significantly higher abundance in both P and Q methods. *Bilophila* sp. was the only member of the phylum *Proteobacteria* that significantly impacted upon variation between methods. Within the phylum *Actinobacteria, Bifidobacterium adolescentis* was reported at significantly higher abundances in M and Z methods. *Bacteroides* sp. was also reported at significantly different levels across the DNA extraction methods.

PERMANOVA pairwise comparisons between the microbial community structures obtained using the HMP and MoBio PowerSoil® DNA Isolation Kit protocols did not suggest they were significantly different. However, analysis of the community structures from the phylum- and genus-level taxa plots revealed minor differences in the recovery of Gram-positive and Gram-negative bacteria. The HMP method recovered significantly higher proportions of Gram-negative *Bacteroidetes, Cyanobacteria* and *Proteobacteria*. The MoBio PowerSoil® DNA Isolation Kit recovered higher proportions of *Actinobacteria* and *Firmicutes*, which are both Gram-positive taxa. It is unclear why the HMP method, which uses the MoBio PowerSoil® DNA Isolation Kit, reported a higher abundance of Gram-negative bacteria, and consequently, lower abundances of Gram-positive bacteria.

### Inter-subject variation

Inter-subject variation contributed the largest proportion of variation (Figure 2, Table S1). Sequencing data from 3 samples, including 45 subsamples, from each subject were combined and are included in analyses of inter-subject variation. Based on relative abundance of taxon-assigned OTUs, members of the bacterial phyla *Bacteroidetes* (Subject 1: 63.4, Subject 2: 73.3, and Subject 3: 75.3%) and *Firmicutes* (26.1, 21.7, and 21.7%) comprised the majority of sequences from each subject's gut microbiota.

A total of 915 OTUs were significantly different between the subjects (*p* < 0.05) in terms of sequence read abundances. The largest proportion of inter-subject variation could be attributed to the increased prevalence of *Prevotella* in Subject 1 (*p* < 10^−25^). Significantly higher proportions of *Ruminococcaceae* (*p* < 10^−25^) and *Cyanobacteria* (*p* < 10^−23^) in Subject 1 were also important drivers of inter-subject variation. Higher proportions of *Bacteroides* sp. observed in Subject 2, and higher proportions of *Sutterella* sp. observed in Subjects 1 and 2, but not Subject 3, were significantly associated with inter-subject variation (*p* < 10^−25^ and <10^−24^, respectively). A significantly higher proportion of *Bacteroides uniformis* was observed in Subject 3 (*p* < 10^−23^).

### Inter-subject variation in new zealand, american, malawi and amerindian populations

**Figure 4:**
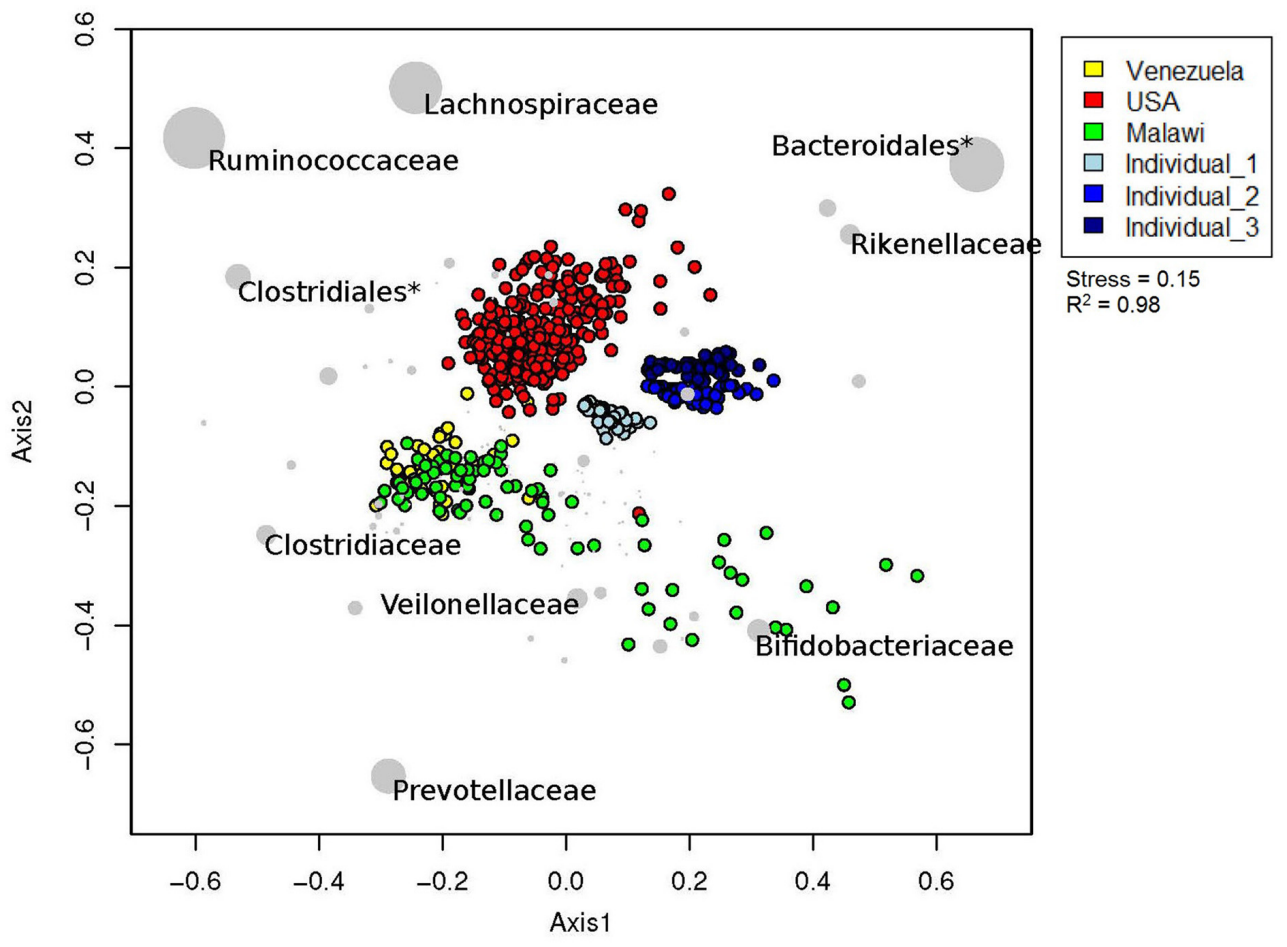
**Relative unweighted UniFrac phylogenetic distances between New Zealand, American, Malawian and Amerindian subjects imaged using two dimensional NMDS plot**. Vectors with a statistically significant contribution to the ordination are overlaid onto the ordination space. The sizes of taxa nodes are based on the average abundances of the taxon in fecal microbial communities from each group of subjects. Asterisk (^*^) denotes a cluster that could not be resolved beyond taxonomic order.

By including our data with those from a larger, international study (Yatsunenko et al., 2012), we were able to test our predictions and initial results regarding inter-subject differences. We removed any subjects <3 years old from the Yatsunenko data set, so that the effect of age on gut microbiota did not affect clustering. The samples clustered primarily by geography/culture (American vs. agrarian vs. New Zealand), and these differences were large enough to outweigh the technical differences observed between the three New Zealand subjects (Figure 4). Notwithstanding the very low numbers of subjects from New Zealand, the 2D NMDS suggested that a transition from western (American and New Zealand) to agrarian (Malawian and Venezuelan) cultures could be associated with an enrichment in members from the bacterial families *Prevotellaceae, Clostridiaceae, Veillonellaceae*, and *Bifidobacteriaceae*. Transitioning from American and agrarian cultures toward New Zealand subjects was associated with an increase in sequences classified to the order *Bacteroidales* and the family *Rikenellaceae* (Figure 4). American subjects were associated with an enrichment in sequences classified to the order *Clostridiales*, and *Ruminococcaceae* and *Lachnospiraceae* families.

Results from ANOSIM tests revealed an overall significant difference in gut microbial community structures between New Zealand, Malawian, American, and Venezuelan countries (Table 3). ANOSIM pairwise tests suggested Malawian and Venezuelan gut community profiles were the most similar, while New Zealand and Venezuelan subjects were the most different. Although the bacterial profiles from New Zealand subjects were significantly different when compared to all other countries, they were most similar and grouped closer to those from America (Figure 4, Table 3).

**Table 3:** **ANOSIM statistics with “BH” adjusted *p*-values on the comparisons between gut microbial community structures of subjects from New Zealand, Malawi, America and Venezuela**.

**Country**	***R*-value**	***p*-value, “BH” adjusted**
New Zealand vs. Malawi vs. America vs. Venezuela	0.797	0.0001
New Zealand vs. Malawi	0.827	0.0001
New Zealand vs. America	0.799	0.0001
New Zealand vs. Venezuela	0.996	0.0001
Malawi vs. America	0.839	0.0001
Malawi vs. Venezuela	0.041	0.1542
America vs. Venezuela	0.815	0.0001

## Discussion

Understanding the origins of variation within an experimental model, and limiting known sources of bias, may help resolve inconsistencies within the literature regarding gut microbial dysbiosis and disease. A recent meta-analysis examined beta diversity among microbial communities generated from human stool samples across several human microbiome studies (Lozupone et al., 2013). Samples clustered strongly by age and geography/culture, which was to be expected (Yatsunenko et al., 2012; Lozupone et al., 2013; Suzuki and Worobey, 2014). However, in data sets comparing fecal samples from subjects with similar ages and geographical/cultural background, secondary clustering by experimental protocol was observed. Our study aimed to identify and quantify the relative contribution of preventable technical bias associated with DNA extraction method.

This study draws attention to the importance of DNA extraction method when interpreting microbial community diversity measurements. Previously, lysis technique has been cited as a contributing factor when extracting microbial nucleic acids (Carrigg et al., 2007; Feinstein et al., 2009). One recent study (Claassen et al., 2013) identified few significant differences between DNA extraction methods when examining lysis technique; however, a different study (Yuan et al., 2012) reported the most effective cell lysis and DNA recovery from bead beating and/or mutanolysin (Yuan et al., 2012; Claassen et al., 2013). In another comparison of lysis techniques, the most effective lysing procedure was hot phenol and bead beating, suggesting that a combination of lysing procedures captures the most accurate community composition (Wu et al., 2010). Our study examined a variety of lysing techniques; however, Shannon and Simpson diversity indices did not reveal significant differences in microbial community diversity specifically pertaining to lysing methods.

The MoBio PowerSoil® DNA Isolation Kit had the second highest median yield of DNA per extraction, and recovered high quality DNA with minimal shearing. While the phenol:chloroform method gave the highest median yield, this result is heavily influenced by the wide range of recovered DNA (7.56–395 ng/μL). Although phenol:chloroform extractions are still widely used, they may take considerably more time than kit methods, and often require extensive clean-up steps before PCR amplification to remove left-over phenol and humic acids. Additionally, human error can affect the reproducibility of these extractions when analysing recovered nucleic acid yield and quality. Compared with the other extraction methods, a large amount of sheared DNA was visualized at the bottom of the phenol:chloroform gel electrophoresis images and DNA yield was not reproducible.

Microbial DNA extracted using the ZR Fecal DNA MiniPrep™ kit returned consistently low DNA yields and poor A260/A280 ratios (<1.8). Analysis of the gel electrophoresis image supports the low amounts of extracted DNA, as bands were barely visible. Results from a study by Claassen et al. indicated much higher amounts of extracted DNA from ZR Fecal DNA MiniPrep™ kit when compared to other DNA extraction methods tested in that study, and when compared to the results presented in our work. Additionally, Claassen et al., reported a substantially higher median quality of DNA extracted using the ZR Fecal DNA MiniPrep™ kit (A260/A280 = 1.678) when compared to the results presented here. Closer examination of the data from that study revealed a substantial range of A260/A280 results below 1.6, even though the results were considered reproducible (CV < 1.0) (Claassen et al., 2013). Significantly lower DNA yield and quality from samples extracted using this kit, and subsequent PCR bias may help explain differences in community structure between ZR Fecal DNA MiniPrep™ and the other methods. Richness and diversity estimates for ZR Fecal DNA MiniPrep™ were significantly higher when compared to the other extraction methods. Claassen et al. (2013) reported the highest Shannon index for the ZR Fecal DNA MiniPrep™ kit, but the value was not significantly different from those estimated for the QIAamp® DNA Stool Mini Kit or MoBio PowerSoil® DNA Isolation Kit. By contrast, the results from our methods comparison suggest the ZR Fecal DNA MiniPrep™ kit was the only method that proved significantly different from the other methods across richness and diversity measures.

Comparisons between the bacterial community profiles resulting from the different DNA extraction methods revealed substantially increased overall bacterial diversity and recovery of members from the *Firmicutes* phylum in the samples extracted using ZR Fecal DNA MiniPrep™. A previous methods study that examined the ZR Fecal DNA MiniPrep™ kit also reported inflated representation of members from the *Firmicutes* phylum, as well as poor DNA quality (Henderson et al., 2013).

The methods examined in this study contribute to the ongoing debate regarding the most accurate and reproducible DNA extraction method. Within the limitations of this study, the results indicate that the most effective microbial DNA extraction method is the MoBio PowerSoil® DNA Isolation Kit. The reproducible high yield and quality of extracted DNA, as well as minimal shearing, support this decision. The community profile was significantly different to that of the ZR Fecal DNA MiniPrep™ kit, however it was similar to the microbial community profiles derived from all other methods. In addition to analyses of biological samples, the inclusion of a mock community, composed of known organisms in known quantities, will help elucidate how technical variation impacts upon recovered microbial community profiles.

The results for biological (inter-subject) vs. technical (DNA extraction method) variation within the New Zealand cohort demonstrate that while DNA extraction does impact upon interpretation of beta diversity, technical variation does not overcome observed community differences between subjects. Furthermore, intra-subject biological variation revealed no significant impact on the observed total variation within the experimental model. These results are consistent with other studies that reported greater inter-subject variation than intra-subject variation for stool samples from adults (Costello et al., 2009; Caporaso et al., 2011; Ursell et al., 2012).

The known, strong effect of inter-subject biological variation, especially related to geography/culture, on the human gut microbiota was observed in the data set presented here. As previously described, the abundance of *Prevotellaceae* was associated with Malawi and Amerindian subjects (Yatsunenko et al., 2012), as well as Subject 1 from New Zealand. Even though significant differences were observed in pairwise ANOSIM tests between subjects from New Zealand, Malawi, America, and Venezuela, the New Zealand subjects were more similar to those from America than the agrarian cultures. A paucity of information exists regarding the New Zealand adult gut microbiota, and the three subjects presented in this study may not be an accurate representation of the average New Zealand gut community. Increasing the number of New Zealand samples may help moderate the extreme biological differences depicted between the three subjects sampled here. Additionally, increasing the number of New Zealand subjects will help describe their gut communities within the global context, especially in relation to other “western” cultures.

High labor costs and time constraints have led to the development of many commercially available DNA extraction kits. Such kits allow researchers to quickly and efficiently extract DNA, with minimal clean-up steps before amplification. While this study is limited by the exclusion of a mock community, or any way of knowing the actual microbial composition within the stool samples, significant differences in community structure were observed between extraction methods, and these differences were the second-greatest contributing factor to variation observed within this study.

### Conflict of interest statement

The authors declare that the research was conducted in the absence of any commercial or financial relationships that could be construed as a potential conflict of interest.
