# Supplementary material for: Evaluating variation in human gut microbiota profiles due to DNA extraction method and inter-subject differences
Source: Front Microbiol. 2015 Feb 18;6:130. doi: 10.3389/fmicb.2015.00130 (PMC4332372; doi:10.3389/fmicb.2015.00130)
Supplement: Supplementary file 1 [file Image1.PDF]

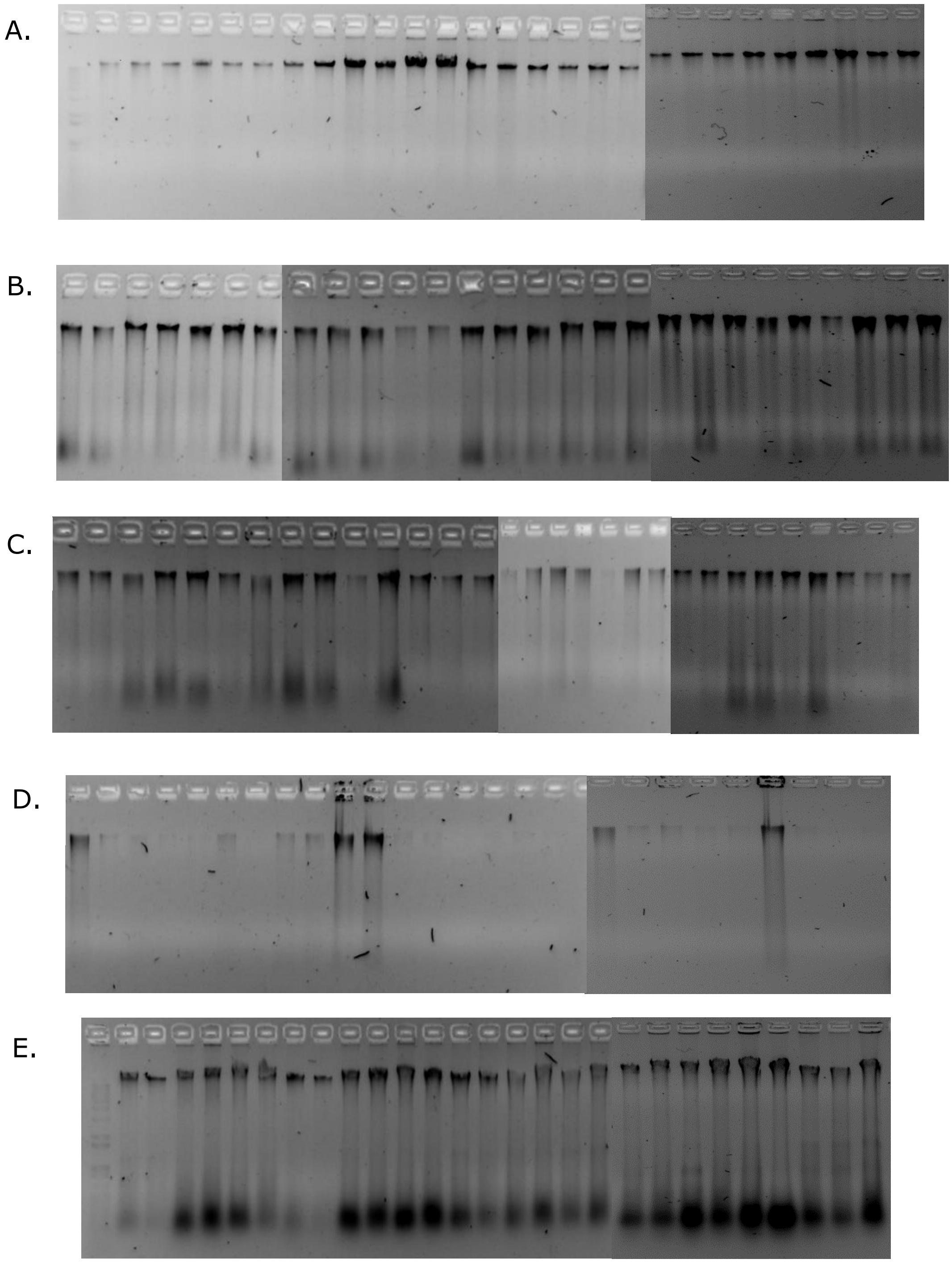

Figure SI1. Integrity of genomic DNA was determined by visualizing 3 μL of extracted DNA on a 1% agarose gel (w/v) containing SYBR Safe DNA Gel Stain (Invitrogen Co., Carlsbad, USA) run in 0.5X TBE buffer at 100 V for 45 min. Shearing of DNA was observed as an increase in smeared, low-density DNA towards the bottom of the gel image. A. Human Microbiome Project method, B. MoBio PowerSoil® DNA Isolation Kit, C. QIAamp® DNA Stool Mini Kit, D. ZR Fecal DNA MiniPrep™, E. phenol:chloroform DNA isolation method.
