# Supplementary material for: Evaluating variation in human gut microbiota profiles due to DNA extraction method and inter-subject differences
Source: Front Microbiol. 2015 Feb 18;6:130. doi: 10.3389/fmicb.2015.00130 (PMC4332372; doi:10.3389/fmicb.2015.00130)
Supplement: Supplementary file 2 [file Table1.DOCX]

Table S1. PERMANOVA results from microbial community profiles rarefied to 11,739 sequences per sample for unweighted UniFrac distance matrix. The R^2^ value can be interpreted as proportion of variation explained (i.e. R^2^ of 0.34= 34% of variation explained). R^2^ values do not necessarily sum to 1 and negative R^2^ values are associated with large *p*-values.

| **Source of Variation** | **d.f.** | **SS** | ***F*** | ***p*-value** | **R^2^** |
| --- | --- | --- | --- | --- | --- |
| Subject | 2 | 11.19 | 16.77 | 0.0001 | 0.34 |
| Extraction Method | 4 | 1.56 | 1.91 | 0.0006 | 0.09 |
| Sample | 2 | 0.34 | 1.01 | 0.4687 | 0.01 |
| Subject x Method | 8 | 1.34 | 1.94 | 0.0001 | 0.09 |
| Subject x Sample | 4 | 0.68 | 1.97 | 0.0001 | 0.07 |
| Method x Sample | 8 | 0.65 | 0.94 | 0.7418 | -0.02 |
| Subject x Sample x Method | 16 | 1.39 | 1.08 | 0.051 | 0.05 |
| Residuals | 90 | 7.25 |  |  | 0.28 |
