# Supplementary material for: Evaluating variation in human gut microbiota profiles due to DNA extraction method and inter-subject differences
Source: Front Microbiol. 2015 Feb 18;6:130. doi: 10.3389/fmicb.2015.00130 (PMC4332372; doi:10.3389/fmicb.2015.00130)
Supplement: Supplementary file 3 [file Table2.DOCX]

Table S2. Relative abundance of taxon-assigned OTUs identified for bacterial and archaeal genera from the stool samples extracted using each method. Each method contains sequencing information from 27 samples. HMP: Human Microbiome Project Extraction Method, MoBio: MoBio PowerSoil® DNA Isolation Kit, P.Chl: phenol:chloroform-based DNA isolation, Qiagen: QIAamp® DNA Stool Mini Kit, Zymo: ZR Fecal DNA MiniPrep™.

| Taxon | HMP | MoBio | P.Chl | Qiagen | Zymo |
| --- | --- | --- | --- | --- | --- |
| Unclassified;Other;Other;Other;Other;Other | 0.006688 | 0.007391 | 0.005835 | 0.007475 | 0.007127 |
| k__Archaea;p__Euryarchaeota;c__Methanobacteria;o__Methanobacteriales;f__Methanobacteriaceae;g__Methanobrevibacter | 6.45E-05 | 7.13E-05 | 0.000183 | 3.66E-05 | 0.000159 |
| k__Bacteria;Other;Other;Other;Other;Other | 0.013367 | 0.013044 | 0.010545 | 0.013402 | 0.012915 |
| k__Bacteria;p__Acidobacteria;c__Solibacteres;o__Solibacterales;f__;g__ | 0 | 0 | 0 | 0 | 6.17E-07 |
| k__Bacteria;p__Actinobacteria;Other;Other;Other;Other | 0 | 0 | 0 | 0 | 6.17E-07 |
| k__Bacteria;p__Actinobacteria;c__Actinobacteria;o__Actinomycetales;f__Actinomycetaceae;g__ | 0 | 0 | 0 | 5.63E-07 | 6.17E-07 |
| k__Bacteria;p__Actinobacteria;c__Actinobacteria;o__Actinomycetales;f__Actinomycetaceae;g__Actinomyces | 2.25E-05 | 4.43E-06 | 5.95E-07 | 1.69E-06 | 2.78E-05 |
| k__Bacteria;p__Actinobacteria;c__Actinobacteria;o__Actinomycetales;f__Actinomycetaceae;g__Mobiluncus | 2.44E-06 | 0 | 0 | 0 | 1.23E-06 |
| k__Bacteria;p__Actinobacteria;c__Actinobacteria;o__Actinomycetales;f__Actinomycetaceae;g__Varibaculum | 2.93E-06 | 4.92E-06 | 5.95E-07 | 1.13E-06 | 1.54E-05 |
| k__Bacteria;p__Actinobacteria;c__Actinobacteria;o__Actinomycetales;f__Corynebacteriaceae;g__Corynebacterium | 4.88E-07 | 4.43E-06 | 2.97E-06 | 2.82E-06 | 7.40E-06 |
| k__Bacteria;p__Actinobacteria;c__Actinobacteria;o__Actinomycetales;f__Micrococcaceae;Other | 0 | 4.92E-07 | 0 | 0 | 0 |
| k__Bacteria;p__Actinobacteria;c__Actinobacteria;o__Actinomycetales;f__Micrococcaceae;g__Rothia | 4.88E-07 | 4.92E-07 | 0 | 2.82E-06 | 8.02E-06 |
| k__Bacteria;p__Actinobacteria;c__Actinobacteria;o__Actinomycetales;f__Propionibacteriaceae;g__ | 0 | 0 | 0 | 0 | 6.17E-07 |
| k__Bacteria;p__Actinobacteria;c__Actinobacteria;o__Bifidobacteriales;f__Bifidobacteriaceae;g__Alloscardovia | 4.88E-07 | 0 | 0 | 0 | 0 |
| k__Bacteria;p__Actinobacteria;c__Actinobacteria;o__Bifidobacteriales;f__Bifidobacteriaceae;g__Bifidobacterium | 0.000789 | 0.002276 | 0.000321 | 0.000269 | 0.00509 |
| k__Bacteria;p__Actinobacteria;c__Actinobacteria;o__Bifidobacteriales;f__Bifidobacteriaceae;g__Scardovia | 0 | 0 | 0 | 5.63E-07 | 1.23E-06 |
| k__Bacteria;p__Actinobacteria;c__Coriobacteriia;o__Coriobacteriales;f__Coriobacteriaceae;Other | 1.81E-05 | 3.89E-05 | 2.68E-05 | 1.24E-05 | 0.000332 |
| k__Bacteria;p__Actinobacteria;c__Coriobacteriia;o__Coriobacteriales;f__Coriobacteriaceae;g__ | 7.81E-05 | 0.000296 | 4.76E-05 | 1.69E-05 | 0.001106 |
| k__Bacteria;p__Actinobacteria;c__Coriobacteriia;o__Coriobacteriales;f__Coriobacteriaceae;g__Adlercreutzia | 6.30E-05 | 9.40E-05 | 6.84E-05 | 4.51E-06 | 0.001679 |
| k__Bacteria;p__Actinobacteria;c__Coriobacteriia;o__Coriobacteriales;f__Coriobacteriaceae;g__Atopobium | 8.30E-06 | 1.48E-06 | 0 | 2.25E-06 | 7.40E-06 |
| k__Bacteria;p__Actinobacteria;c__Coriobacteriia;o__Coriobacteriales;f__Coriobacteriaceae;g__Collinsella | 0.000115 | 0.00019 | 1.01E-05 | 3.04E-05 | 0.000255 |
| k__Bacteria;p__Actinobacteria;c__Coriobacteriia;o__Coriobacteriales;f__Coriobacteriaceae;g__Eggerthella | 8.30E-06 | 1.72E-05 | 1.78E-06 | 1.69E-06 | 5.86E-05 |
| k__Bacteria;p__Actinobacteria;c__Coriobacteriia;o__Coriobacteriales;f__Coriobacteriaceae;g__Slackia | 0 | 0 | 0 | 0 | 6.17E-07 |
| k__Bacteria;p__Actinobacteria;c__Thermoleophilia;o__Gaiellales;f__Gaiellaceae;g__ | 0 | 0 | 0 | 0 | 6.17E-07 |
| k__Bacteria;p__Bacteroidetes;Other;Other;Other;Other | 0 | 1.48E-06 | 0 | 1.69E-06 | 6.17E-07 |
| k__Bacteria;p__Bacteroidetes;c__;o__;f__;g__ | 0 | 0 | 0 | 0 | 6.17E-07 |
| k__Bacteria;p__Bacteroidetes;c__Bacteroidia;o__Bacteroidales;Other;Other | 0.000141 | 7.33E-05 | 8.74E-05 | 0.000122 | 3.52E-05 |
| k__Bacteria;p__Bacteroidetes;c__Bacteroidia;o__Bacteroidales;f__;g__ | 0 | 3.44E-06 | 0 | 5.63E-07 | 6.17E-07 |
| k__Bacteria;p__Bacteroidetes;c__Bacteroidia;o__Bacteroidales;f__Bacteroidaceae;Other | 4.88E-06 | 6.39E-06 | 6.54E-06 | 4.51E-06 | 1.23E-06 |
| k__Bacteria;p__Bacteroidetes;c__Bacteroidia;o__Bacteroidales;f__Bacteroidaceae;g__Bacteroides | 0.513656 | 0.452942 | 0.486906 | 0.510049 | 0.290217 |
| k__Bacteria;p__Bacteroidetes;c__Bacteroidia;o__Bacteroidales;f__Porphyromonadaceae;Other | 1.27E-05 | 3.94E-06 | 5.95E-06 | 8.45E-06 | 5.55E-06 |
| k__Bacteria;p__Bacteroidetes;c__Bacteroidia;o__Bacteroidales;f__Porphyromonadaceae;g__Parabacteroides | 0.038422 | 0.011829 | 0.022998 | 0.030927 | 0.010194 |
| k__Bacteria;p__Bacteroidetes;c__Bacteroidia;o__Bacteroidales;f__Porphyromonadaceae;g__Porphyromonas | 5.37E-05 | 2.71E-05 | 1.90E-05 | 2.65E-05 | 2.28E-05 |
| k__Bacteria;p__Bacteroidetes;c__Bacteroidia;o__Bacteroidales;f__Prevotellaceae;g__Prevotella | 0.090394 | 0.205188 | 0.189347 | 0.169907 | 0.079642 |
| k__Bacteria;p__Bacteroidetes;c__Bacteroidia;o__Bacteroidales;f__Rikenellaceae;Other | 4.40E-06 | 4.92E-07 | 5.95E-07 | 1.13E-06 | 0 |
| k__Bacteria;p__Bacteroidetes;c__Bacteroidia;o__Bacteroidales;f__Rikenellaceae;g__ | 0.071951 | 0.054729 | 0.042006 | 0.040268 | 0.04583 |
| k__Bacteria;p__Bacteroidetes;c__Bacteroidia;o__Bacteroidales;f__S24-7;g__ | 0.00049 | 0.000214 | 0.0003 | 0.000444 | 0.00013 |
| k__Bacteria;p__Bacteroidetes;c__Bacteroidia;o__Bacteroidales;f__[Barnesiellaceae];g__ | 0.021971 | 0.029404 | 0.021291 | 0.020985 | 0.020526 |
| k__Bacteria;p__Bacteroidetes;c__Bacteroidia;o__Bacteroidales;f__[Odoribacteraceae];g__Butyricimonas | 0.001869 | 0.002129 | 0.001453 | 0.001074 | 0.001006 |
| k__Bacteria;p__Bacteroidetes;c__Bacteroidia;o__Bacteroidales;f__[Odoribacteraceae];g__Odoribacter | 0.003952 | 0.006375 | 0.003955 | 0.003637 | 0.004173 |
| k__Bacteria;p__Chlorobi;c__OPB56;o__;f__;g__ | 0 | 0 | 0 | 0 | 6.17E-07 |
| k__Bacteria;p__Cyanobacteria;Other;Other;Other;Other | 1.95E-06 | 1.97E-06 | 0 | 5.63E-07 | 0 |
| k__Bacteria;p__Cyanobacteria;c__4C0d-2;o__YS2;f__;g__ | 0.050753 | 0.011271 | 0.003651 | 0.003697 | 0.003665 |
| k__Bacteria;p__Cyanobacteria;c__Chloroplast;o__Streptophyta;f__;g__ | 2.30E-05 | 8.85E-06 | 1.78E-06 | 5.18E-05 | 0.000101 |
| k__Bacteria;p__Firmicutes;Other;Other;Other;Other | 0.000839 | 0.000463 | 0.00037 | 0.000244 | 0.000736 |
| k__Bacteria;p__Firmicutes;c__Bacilli;o__Bacillales;f__Alicyclobacillaceae;g__Alicyclobacillus | 0 | 0 | 0 | 0 | 1.23E-06 |
| k__Bacteria;p__Firmicutes;c__Bacilli;o__Bacillales;f__Paenibacillaceae;g__Paenibacillus | 4.88E-07 | 9.84E-07 | 5.95E-07 | 0 | 4.93E-06 |
| k__Bacteria;p__Firmicutes;c__Bacilli;o__Bacillales;f__Planococcaceae;Other | 0 | 4.92E-07 | 0 | 0 | 5.55E-06 |
| k__Bacteria;p__Firmicutes;c__Bacilli;o__Bacillales;f__Planococcaceae;g__Sporosarcina | 0 | 0 | 0 | 1.13E-06 | 0 |
| k__Bacteria;p__Firmicutes;c__Bacilli;o__Bacillales;f__Staphylococcaceae;g__Staphylococcus | 4.88E-07 | 0 | 0 | 1.13E-06 | 0 |
| k__Bacteria;p__Firmicutes;c__Bacilli;o__Gemellales;f__Gemellaceae;g__ | 4.40E-06 | 2.95E-06 | 0 | 4.51E-06 | 1.54E-05 |
| k__Bacteria;p__Firmicutes;c__Bacilli;o__Lactobacillales;Other;Other | 1.95E-06 | 9.84E-07 | 5.95E-07 | 5.63E-07 | 2.16E-05 |
| k__Bacteria;p__Firmicutes;c__Bacilli;o__Lactobacillales;f__Aerococcaceae;g__ | 0 | 0 | 1.19E-06 | 0 | 0 |
| k__Bacteria;p__Firmicutes;c__Bacilli;o__Lactobacillales;f__Aerococcaceae;g__Abiotrophia | 0 | 4.92E-07 | 0 | 0 | 0 |
| k__Bacteria;p__Firmicutes;c__Bacilli;o__Lactobacillales;f__Carnobacteriaceae;g__Granulicatella | 6.35E-06 | 3.94E-06 | 0 | 1.13E-06 | 3.89E-05 |
| k__Bacteria;p__Firmicutes;c__Bacilli;o__Lactobacillales;f__Enterococcaceae;g__Enterococcus | 4.88E-07 | 1.97E-06 | 1.19E-05 | 1.69E-06 | 6.48E-05 |
| k__Bacteria;p__Firmicutes;c__Bacilli;o__Lactobacillales;f__Lactobacillaceae;Other | 0 | 4.92E-06 | 1.78E-06 | 5.63E-07 | 8.45E-05 |
| k__Bacteria;p__Firmicutes;c__Bacilli;o__Lactobacillales;f__Lactobacillaceae;g__ | 0 | 0 | 0 | 0 | 3.70E-06 |
| k__Bacteria;p__Firmicutes;c__Bacilli;o__Lactobacillales;f__Lactobacillaceae;g__Lactobacillus | 9.77E-07 | 2.95E-06 | 1.19E-06 | 2.25E-06 | 2.90E-05 |
| k__Bacteria;p__Firmicutes;c__Bacilli;o__Lactobacillales;f__Lactobacillaceae;g__Pediococcus | 2.44E-06 | 4.18E-05 | 3.69E-05 | 2.82E-06 | 0.000797 |
| k__Bacteria;p__Firmicutes;c__Bacilli;o__Lactobacillales;f__Leuconostocaceae;Other | 0 | 1.48E-06 | 5.95E-07 | 0 | 8.63E-06 |
| k__Bacteria;p__Firmicutes;c__Bacilli;o__Lactobacillales;f__Leuconostocaceae;g__Leuconostoc | 3.91E-06 | 1.97E-06 | 0 | 5.63E-07 | 6.17E-06 |
| k__Bacteria;p__Firmicutes;c__Bacilli;o__Lactobacillales;f__Streptococcaceae;g__Lactococcus | 1.12E-05 | 2.21E-05 | 6.54E-06 | 0.000265 | 0.000389 |
| k__Bacteria;p__Firmicutes;c__Bacilli;o__Lactobacillales;f__Streptococcaceae;g__Streptococcus | 0.000137 | 0.000257 | 0.000125 | 0.000174 | 0.002217 |
| k__Bacteria;p__Firmicutes;c__Bacilli;o__Turicibacterales;f__Turicibacteraceae;g__Turicibacter | 0.001027 | 0.000192 | 0.000199 | 0.000189 | 0.000398 |
| k__Bacteria;p__Firmicutes;c__Clostridia;Other;Other;Other | 2.54E-05 | 1.72E-05 | 2.14E-05 | 2.20E-05 | 0.00012 |
| k__Bacteria;p__Firmicutes;c__Clostridia;o__Clostridiales;Other;Other | 0.001486 | 0.00306 | 0.002104 | 0.002633 | 0.009968 |
| k__Bacteria;p__Firmicutes;c__Clostridia;o__Clostridiales;f__;g__ | 0.012276 | 0.008486 | 0.008458 | 0.006935 | 0.010199 |
| k__Bacteria;p__Firmicutes;c__Clostridia;o__Clostridiales;f__Christensenellaceae;Other | 0 | 4.92E-07 | 5.95E-07 | 1.13E-06 | 3.08E-06 |
| k__Bacteria;p__Firmicutes;c__Clostridia;o__Clostridiales;f__Christensenellaceae;g__ | 4.30E-05 | 0.000102 | 5.95E-05 | 0.000108 | 0.000175 |
| k__Bacteria;p__Firmicutes;c__Clostridia;o__Clostridiales;f__Christensenellaceae;g__Christensenella | 4.40E-06 | 8.36E-06 | 6.54E-06 | 1.13E-06 | 5.43E-05 |
| k__Bacteria;p__Firmicutes;c__Clostridia;o__Clostridiales;f__Clostridiaceae;Other | 0.000725 | 0.000487 | 0.000213 | 0.00017 | 0.001844 |
| k__Bacteria;p__Firmicutes;c__Clostridia;o__Clostridiales;f__Clostridiaceae;g__ | 1.90E-05 | 0.000205 | 1.72E-05 | 3.49E-05 | 0.000369 |
| k__Bacteria;p__Firmicutes;c__Clostridia;o__Clostridiales;f__Clostridiaceae;g__02d06 | 2.98E-05 | 8.17E-05 | 9.99E-05 | 0.000174 | 3.70E-05 |
| k__Bacteria;p__Firmicutes;c__Clostridia;o__Clostridiales;f__Clostridiaceae;g__Clostridium | 0.001004 | 0.001048 | 0.000369 | 0.000583 | 0.003876 |
| k__Bacteria;p__Firmicutes;c__Clostridia;o__Clostridiales;f__Clostridiaceae;g__SMB53 | 0.000656 | 0.000751 | 0.000357 | 0.000252 | 0.010092 |
| k__Bacteria;p__Firmicutes;c__Clostridia;o__Clostridiales;f__Dehalobacteriaceae;g__ | 9.77E-07 | 1.97E-06 | 2.38E-06 | 1.69E-06 | 4.93E-06 |
| k__Bacteria;p__Firmicutes;c__Clostridia;o__Clostridiales;f__Dehalobacteriaceae;g__Dehalobacterium | 7.33E-06 | 5.41E-06 | 1.37E-05 | 3.38E-06 | 7.40E-06 |
| k__Bacteria;p__Firmicutes;c__Clostridia;o__Clostridiales;f__Eubacteriaceae;g__Anaerofustis | 1.95E-06 | 5.90E-06 | 5.95E-07 | 1.13E-06 | 1.60E-05 |
| k__Bacteria;p__Firmicutes;c__Clostridia;o__Clostridiales;f__Eubacteriaceae;g__Pseudoramibacter_Eubacterium | 0 | 4.92E-06 | 5.95E-07 | 0 | 2.10E-05 |
| k__Bacteria;p__Firmicutes;c__Clostridia;o__Clostridiales;f__Lachnospiraceae;Other | 0.005481 | 0.010214 | 0.0063 | 0.009692 | 0.028727 |
| k__Bacteria;p__Firmicutes;c__Clostridia;o__Clostridiales;f__Lachnospiraceae;g__ | 0.007216 | 0.016832 | 0.024708 | 0.024311 | 0.023251 |
| k__Bacteria;p__Firmicutes;c__Clostridia;o__Clostridiales;f__Lachnospiraceae;g__Anaerostipes | 0.000561 | 0.000846 | 0.000356 | 0.000933 | 0.003782 |
| k__Bacteria;p__Firmicutes;c__Clostridia;o__Clostridiales;f__Lachnospiraceae;g__Blautia | 0.003178 | 0.009361 | 0.002827 | 0.001255 | 0.044551 |
| k__Bacteria;p__Firmicutes;c__Clostridia;o__Clostridiales;f__Lachnospiraceae;g__Coprococcus | 0.005281 | 0.014069 | 0.004057 | 0.00323 | 0.090524 |
| k__Bacteria;p__Firmicutes;c__Clostridia;o__Clostridiales;f__Lachnospiraceae;g__Dorea | 0.000333 | 0.001281 | 0.000442 | 0.000495 | 0.006372 |
| k__Bacteria;p__Firmicutes;c__Clostridia;o__Clostridiales;f__Lachnospiraceae;g__Epulopiscium | 2.44E-06 | 0 | 0 | 5.63E-07 | 3.70E-06 |
| k__Bacteria;p__Firmicutes;c__Clostridia;o__Clostridiales;f__Lachnospiraceae;g__Lachnobacterium | 3.52E-05 | 8.56E-05 | 0.000197 | 8.28E-05 | 0.000192 |
| k__Bacteria;p__Firmicutes;c__Clostridia;o__Clostridiales;f__Lachnospiraceae;g__Lachnospira | 0.003458 | 0.00447 | 0.008648 | 0.016959 | 0.012528 |
| k__Bacteria;p__Firmicutes;c__Clostridia;o__Clostridiales;f__Lachnospiraceae;g__Moryella | 0 | 0 | 0 | 5.63E-07 | 1.23E-06 |
| k__Bacteria;p__Firmicutes;c__Clostridia;o__Clostridiales;f__Lachnospiraceae;g__Oribacterium | 0 | 4.92E-07 | 0 | 1.13E-06 | 8.63E-06 |
| k__Bacteria;p__Firmicutes;c__Clostridia;o__Clostridiales;f__Lachnospiraceae;g__Pseudobutyrivibrio | 0 | 0 | 0 | 0 | 6.17E-07 |
| k__Bacteria;p__Firmicutes;c__Clostridia;o__Clostridiales;f__Lachnospiraceae;g__Roseburia | 0.008679 | 0.013644 | 0.012357 | 0.010242 | 0.026093 |
| k__Bacteria;p__Firmicutes;c__Clostridia;o__Clostridiales;f__Lachnospiraceae;g__[Ruminococcus] | 0.001645 | 0.004114 | 0.001942 | 0.001793 | 0.035533 |
| k__Bacteria;p__Firmicutes;c__Clostridia;o__Clostridiales;f__Peptostreptococcaceae;g__ | 2.44E-06 | 9.35E-06 | 1.78E-06 | 1.13E-06 | 7.34E-05 |
| k__Bacteria;p__Firmicutes;c__Clostridia;o__Clostridiales;f__Peptostreptococcaceae;g__Peptostreptococcus | 2.44E-06 | 0 | 0 | 5.63E-07 | 3.08E-06 |
| k__Bacteria;p__Firmicutes;c__Clostridia;o__Clostridiales;f__Ruminococcaceae;Other | 0.011348 | 0.011825 | 0.018209 | 0.014524 | 0.021487 |
| k__Bacteria;p__Firmicutes;c__Clostridia;o__Clostridiales;f__Ruminococcaceae;g__ | 0.002345 | 0.003493 | 0.002532 | 0.001951 | 0.004081 |
| k__Bacteria;p__Firmicutes;c__Clostridia;o__Clostridiales;f__Ruminococcaceae;g__Anaerofilum | 1.47E-06 | 4.43E-06 | 5.95E-06 | 3.94E-06 | 3.08E-06 |
| k__Bacteria;p__Firmicutes;c__Clostridia;o__Clostridiales;f__Ruminococcaceae;g__Anaerotruncus | 1.86E-05 | 2.21E-05 | 7.73E-06 | 2.48E-05 | 5.92E-05 |
| k__Bacteria;p__Firmicutes;c__Clostridia;o__Clostridiales;f__Ruminococcaceae;g__Faecalibacterium | 0.021533 | 0.042689 | 0.06558 | 0.023361 | 0.087338 |
| k__Bacteria;p__Firmicutes;c__Clostridia;o__Clostridiales;f__Ruminococcaceae;g__Oscillospira | 0.026353 | 0.017505 | 0.019204 | 0.025104 | 0.040003 |
| k__Bacteria;p__Firmicutes;c__Clostridia;o__Clostridiales;f__Ruminococcaceae;g__Ruminococcus | 0.00843 | 0.016472 | 0.004274 | 0.007576 | 0.023269 |
| k__Bacteria;p__Firmicutes;c__Clostridia;o__Clostridiales;f__Veillonellaceae;Other | 1.07E-05 | 1.48E-06 | 1.19E-06 | 6.20E-06 | 1.97E-05 |
| k__Bacteria;p__Firmicutes;c__Clostridia;o__Clostridiales;f__Veillonellaceae;g__Dialister | 0.00041 | 0.000958 | 0.00159 | 0.003046 | 0.002217 |
| k__Bacteria;p__Firmicutes;c__Clostridia;o__Clostridiales;f__Veillonellaceae;g__Megasphaera | 0 | 4.92E-07 | 0 | 0 | 0 |
| k__Bacteria;p__Firmicutes;c__Clostridia;o__Clostridiales;f__Veillonellaceae;g__Phascolarctobacterium | 0.00179 | 0.003835 | 0.009845 | 0.01912 | 0.005066 |
| k__Bacteria;p__Firmicutes;c__Clostridia;o__Clostridiales;f__Veillonellaceae;g__Schwartzia | 9.77E-07 | 0 | 0 | 0 | 0 |
| k__Bacteria;p__Firmicutes;c__Clostridia;o__Clostridiales;f__Veillonellaceae;g__Veillonella | 0.00073 | 0.000968 | 0.001023 | 0.001431 | 0.002163 |
| k__Bacteria;p__Firmicutes;c__Clostridia;o__Clostridiales;f__[Mogibacteriaceae];Other | 1.47E-06 | 4.92E-07 | 0 | 0 | 3.70E-06 |
| k__Bacteria;p__Firmicutes;c__Clostridia;o__Clostridiales;f__[Mogibacteriaceae];g__ | 0.000187 | 0.000305 | 0.000262 | 0.000197 | 0.00125 |
| k__Bacteria;p__Firmicutes;c__Clostridia;o__Clostridiales;f__[Mogibacteriaceae];g__Mogibacterium | 4.88E-07 | 0 | 0 | 5.63E-07 | 0 |
| k__Bacteria;p__Firmicutes;c__Clostridia;o__Clostridiales;f__[Tissierellaceae];g__ | 0 | 0 | 0 | 0 | 6.17E-07 |
| k__Bacteria;p__Firmicutes;c__Clostridia;o__Clostridiales;f__[Tissierellaceae];g__1-68 | 2.44E-06 | 1.48E-06 | 2.38E-06 | 6.76E-06 | 3.70E-06 |
| k__Bacteria;p__Firmicutes;c__Clostridia;o__Clostridiales;f__[Tissierellaceae];g__Anaerococcus | 1.47E-06 | 9.84E-07 | 0 | 1.13E-06 | 3.08E-06 |
| k__Bacteria;p__Firmicutes;c__Clostridia;o__Clostridiales;f__[Tissierellaceae];g__Finegoldia | 4.88E-07 | 0 | 0 | 0 | 6.17E-07 |
| k__Bacteria;p__Firmicutes;c__Clostridia;o__Clostridiales;f__[Tissierellaceae];g__Parvimonas | 0 | 0 | 0 | 0 | 6.17E-06 |
| k__Bacteria;p__Firmicutes;c__Clostridia;o__Clostridiales;f__[Tissierellaceae];g__Peptoniphilus | 3.42E-06 | 0 | 2.38E-06 | 5.07E-06 | 4.32E-06 |
| k__Bacteria;p__Firmicutes;c__Clostridia;o__Clostridiales;f__[Tissierellaceae];g__WAL_1855D | 1.27E-05 | 4.92E-06 | 1.25E-05 | 3.44E-05 | 1.54E-05 |
| k__Bacteria;p__Firmicutes;c__Clostridia;o__Clostridiales;f__[Tissierellaceae];g__ph2 | 4.88E-07 | 4.92E-07 | 0 | 1.13E-06 | 0 |
| k__Bacteria;p__Firmicutes;c__Erysipelotrichi;o__Erysipelotrichales;f__Erysipelotrichaceae;Other | 2.44E-05 | 1.92E-05 | 7.73E-06 | 3.38E-06 | 5.55E-05 |
| k__Bacteria;p__Firmicutes;c__Erysipelotrichi;o__Erysipelotrichales;f__Erysipelotrichaceae;g__ | 0.000336 | 0.000868 | 0.000171 | 0.000229 | 0.005161 |
| k__Bacteria;p__Firmicutes;c__Erysipelotrichi;o__Erysipelotrichales;f__Erysipelotrichaceae;g__Bulleidia | 2.44E-06 | 0 | 0 | 5.63E-07 | 3.70E-06 |
| k__Bacteria;p__Firmicutes;c__Erysipelotrichi;o__Erysipelotrichales;f__Erysipelotrichaceae;g__Coprobacillus | 2.25E-05 | 1.57E-05 | 1.43E-05 | 7.89E-06 | 0.001074 |
| k__Bacteria;p__Firmicutes;c__Erysipelotrichi;o__Erysipelotrichales;f__Erysipelotrichaceae;g__Holdemania | 1.71E-05 | 2.21E-05 | 2.56E-05 | 2.82E-05 | 2.71E-05 |
| k__Bacteria;p__Firmicutes;c__Erysipelotrichi;o__Erysipelotrichales;f__Erysipelotrichaceae;g__[Eubacterium] | 0.000102 | 0.000494 | 0.00013 | 0.000141 | 0.001009 |
| k__Bacteria;p__Firmicutes;c__Erysipelotrichi;o__Erysipelotrichales;f__Erysipelotrichaceae;g__cc_115 | 9.77E-06 | 3.30E-05 | 3.57E-06 | 3.94E-06 | 2.22E-05 |
| k__Bacteria;p__Fusobacteria;c__Fusobacteriia;o__Fusobacteriales;f__Fusobacteriaceae;g__Fusobacterium | 9.77E-07 | 4.92E-07 | 0 | 2.82E-06 | 0 |
| k__Bacteria;p__Lentisphaerae;c__[Lentisphaeria];o__Victivallales;f__Victivallaceae;g__ | 0.000342 | 4.57E-05 | 5.95E-06 | 1.30E-05 | 2.47E-06 |
| k__Bacteria;p__Proteobacteria;Other;Other;Other;Other | 0 | 0 | 5.95E-07 | 0 | 6.17E-07 |
| k__Bacteria;p__Proteobacteria;c__Alphaproteobacteria;Other;Other;Other | 4.88E-07 | 4.92E-07 | 0 | 5.63E-07 | 0 |
| k__Bacteria;p__Proteobacteria;c__Alphaproteobacteria;o__RF32;f__;g__ | 0.006542 | 0.000211 | 7.02E-05 | 0.000372 | 0.000184 |
| k__Bacteria;p__Proteobacteria;c__Alphaproteobacteria;o__Rhizobiales;f__;g__ | 0 | 0 | 0 | 0 | 6.17E-07 |
| k__Bacteria;p__Proteobacteria;c__Alphaproteobacteria;o__Rhizobiales;f__Aurantimonadaceae;g__ | 0 | 0 | 0 | 0 | 4.32E-06 |
| k__Bacteria;p__Proteobacteria;c__Alphaproteobacteria;o__Rhizobiales;f__Hyphomicrobiaceae;g__Rhodoplanes | 0 | 0 | 0 | 0 | 6.17E-07 |
| k__Bacteria;p__Proteobacteria;c__Alphaproteobacteria;o__Rhodospirillales;f__Rhodospirillaceae;Other | 0 | 0 | 0 | 0 | 6.17E-07 |
| k__Bacteria;p__Proteobacteria;c__Alphaproteobacteria;o__Rhodospirillales;f__Rhodospirillaceae;g__ | 0 | 0 | 0 | 0 | 6.17E-07 |
| k__Bacteria;p__Proteobacteria;c__Alphaproteobacteria;o__Rickettsiales;f__mitochondria;g__ | 1.47E-06 | 4.92E-07 | 0 | 5.63E-07 | 6.17E-07 |
| k__Bacteria;p__Proteobacteria;c__Alphaproteobacteria;o__Sphingomonadales;f__Sphingomonadaceae;g__Sphingobium | 0 | 0 | 0 | 1.13E-06 | 0 |
| k__Bacteria;p__Proteobacteria;c__Alphaproteobacteria;o__Sphingomonadales;f__Sphingomonadaceae;g__Sphingomonas | 0 | 0 | 0 | 3.38E-06 | 6.17E-07 |
| k__Bacteria;p__Proteobacteria;c__Betaproteobacteria;Other;Other;Other | 0.000832 | 7.62E-05 | 3.63E-05 | 9.13E-05 | 6.11E-05 |
| k__Bacteria;p__Proteobacteria;c__Betaproteobacteria;o__Burkholderiales;Other;Other | 0.000159 | 2.12E-05 | 1.61E-05 | 1.69E-05 | 9.87E-06 |
| k__Bacteria;p__Proteobacteria;c__Betaproteobacteria;o__Burkholderiales;f__Alcaligenaceae;Other | 2.10E-05 | 6.89E-06 | 3.57E-06 | 1.13E-06 | 1.23E-06 |
| k__Bacteria;p__Proteobacteria;c__Betaproteobacteria;o__Burkholderiales;f__Alcaligenaceae;g__Sutterella | 0.042741 | 0.006804 | 0.006227 | 0.011459 | 0.004192 |
| k__Bacteria;p__Proteobacteria;c__Betaproteobacteria;o__Burkholderiales;f__Burkholderiaceae;g__Lautropia | 0 | 4.92E-07 | 0 | 0 | 0 |
| k__Bacteria;p__Proteobacteria;c__Betaproteobacteria;o__Burkholderiales;f__Comamonadaceae;g__Comamonas | 0 | 0 | 0 | 0 | 1.23E-06 |
| k__Bacteria;p__Proteobacteria;c__Betaproteobacteria;o__Burkholderiales;f__Oxalobacteraceae;g__Cupriavidus | 0 | 0 | 0 | 5.63E-07 | 0 |
| k__Bacteria;p__Proteobacteria;c__Betaproteobacteria;o__Burkholderiales;f__Oxalobacteraceae;g__Janthinobacterium | 0 | 0 | 0 | 1.13E-06 | 6.17E-07 |
| k__Bacteria;p__Proteobacteria;c__Betaproteobacteria;o__Burkholderiales;f__Oxalobacteraceae;g__Oxalobacter | 4.35E-05 | 0.000128 | 0.000122 | 0.00014 | 0.000117 |
| k__Bacteria;p__Proteobacteria;c__Betaproteobacteria;o__Neisseriales;f__Neisseriaceae;g__ | 0 | 0 | 1.78E-06 | 0 | 0 |
| k__Bacteria;p__Proteobacteria;c__Betaproteobacteria;o__Neisseriales;f__Neisseriaceae;g__Neisseria | 0 | 0 | 0 | 2.82E-06 | 0 |
| k__Bacteria;p__Proteobacteria;c__Deltaproteobacteria;o__Desulfovibrionales;Other;Other | 0 | 4.92E-07 | 0 | 0 | 6.17E-07 |
| k__Bacteria;p__Proteobacteria;c__Deltaproteobacteria;o__Desulfovibrionales;f__Desulfovibrionaceae;g__ | 6.40E-05 | 0.000208 | 0.000249 | 0.00027 | 0.000102 |
| k__Bacteria;p__Proteobacteria;c__Deltaproteobacteria;o__Desulfovibrionales;f__Desulfovibrionaceae;g__Bilophila | 0.001944 | 0.001249 | 0.004166 | 0.003545 | 0.001575 |
| k__Bacteria;p__Proteobacteria;c__Epsilonproteobacteria;o__Campylobacterales;f__Campylobacteraceae;g__Campylobacter | 6.84E-06 | 9.35E-06 | 7.13E-06 | 9.02E-06 | 1.05E-05 |
| k__Bacteria;p__Proteobacteria;c__Gammaproteobacteria;o__Alteromonadales;f__Shewanellaceae;g__Shewanella | 0 | 0 | 0 | 0 | 6.17E-07 |
| k__Bacteria;p__Proteobacteria;c__Gammaproteobacteria;o__Enterobacteriales;f__Enterobacteriaceae;Other | 0.000299 | 0.001199 | 0.00118 | 0.001873 | 0.000404 |
| k__Bacteria;p__Proteobacteria;c__Gammaproteobacteria;o__Enterobacteriales;f__Enterobacteriaceae;g__Salmonella | 0 | 0 | 0 | 0 | 6.17E-07 |
| k__Bacteria;p__Proteobacteria;c__Gammaproteobacteria;o__Pasteurellales;f__Pasteurellaceae;Other | 6.35E-06 | 3.94E-06 | 0 | 1.69E-06 | 3.70E-06 |
| k__Bacteria;p__Proteobacteria;c__Gammaproteobacteria;o__Pasteurellales;f__Pasteurellaceae;g__Aggregatibacter | 3.22E-05 | 4.28E-05 | 2.85E-05 | 4.56E-05 | 1.97E-05 |
| k__Bacteria;p__Proteobacteria;c__Gammaproteobacteria;o__Pasteurellales;f__Pasteurellaceae;g__Haemophilus | 0.000624 | 0.000684 | 0.000128 | 0.00074 | 0.000252 |
| k__Bacteria;p__Proteobacteria;c__Gammaproteobacteria;o__Pseudomonadales;f__Moraxellaceae;g__Acinetobacter | 0 | 0 | 2.97E-06 | 0 | 1.85E-06 |
| k__Bacteria;p__Proteobacteria;c__Gammaproteobacteria;o__Pseudomonadales;f__Pseudomonadaceae;g__Pseudomonas | 1.95E-06 | 2.46E-06 | 2.38E-06 | 1.52E-05 | 1.85E-06 |
| k__Bacteria;p__Synergistetes;c__Synergistia;o__Synergistales;f__Dethiosulfovibrionaceae;g__Pyramidobacter | 4.88E-07 | 9.84E-07 | 0 | 2.82E-06 | 6.17E-07 |
| k__Bacteria;p__Synergistetes;c__Synergistia;o__Synergistales;f__Dethiosulfovibrionaceae;g__TG5 | 0 | 0 | 0 | 5.63E-07 | 0 |
| k__Bacteria;p__TM7;c__TM7-3;o__;f__;g__ | 9.77E-07 | 0 | 0 | 0 | 0 |
| k__Bacteria;p__Tenericutes;Other;Other;Other;Other | 0 | 0 | 0 | 5.63E-07 | 0 |
| k__Bacteria;p__Tenericutes;c__Mollicutes;o__RF39;f__;g__ | 0.00148 | 0.001667 | 0.000903 | 0.00105 | 0.00088 |
| k__Bacteria;p__Tenericutes;c__RF3;o__ML615J-28;f__;g__ | 0.001297 | 0.000144 | 5.41E-05 | 8.23E-05 | 8.14E-05 |
| k__Bacteria;p__Verrucomicrobia;c__Verrucomicrobiae;o__Verrucomicrobiales;f__Verrucomicrobiaceae;g__Akkermansia | 0.000749 | 0.000124 | 0.00051 | 0.000181 | 0.000138 |
| k__Bacteria;p__[Thermi];c__Deinococci;o__Deinococcales;f__Deinococcaceae;g__Deinococcus | 0 | 0 | 0 | 0 | 6.17E-07 |
| k__Bacteria;p__[Thermi];c__Deinococci;o__Thermales;f__Thermaceae;g__Meiothermus | 0 | 0 | 0 | 0 | 6.17E-07 |
| k__Bacteria;p__[Thermi];c__Deinococci;o__Thermales;f__Thermaceae;g__Thermus | 0 | 0 | 0 | 0 | 8.02E-06 |
