# Supplementary material for: Evaluating variation in human gut microbiota profiles due to DNA extraction method and inter-subject differences
Source: Front Microbiol. 2015 Feb 18;6:130. doi: 10.3389/fmicb.2015.00130 (PMC4332372; doi:10.3389/fmicb.2015.00130)
Supplement: Supplementary file 4 [file Table3.DOCX]

Table S3. Group statistics comparing mean values of taxon-classified OTUs from each method (n=27 per method). Significant differences (*p* < 0.05, Bonferroni corrected) of OTU abundances between methods are shown.

| OTU | Test-Statistic | P | FDR_P | Bonferroni_P | P _mean | Q_mean | Z_mean | HMP_mean | M_mean | taxonomy |
| --- | --- | --- | --- | --- | --- | --- | --- | --- | --- | --- |
| OTU_29 | 100.8300024 | 6.55E-21 | 1.35E-17 | 2.29E-17 | 7.518518519 | 2.296296296 | 229.9259259 | 12.11111111 | 46.77777778 | k__Bacteria;p__Firmicutes;c__Clostridia;o__Clostridiales;f__Lachnospiraceae;g__Blautia;s__ |
| OTU_38 | 100.493347 | 7.72E-21 | 1.35E-17 | 2.70E-17 | 2.407407407 | 0.333333333 | 120 | 6.962962963 | 15.81481481 | k__Bacteria;p__Firmicutes;c__Clostridia;o__Clostridiales;f__Lachnospiraceae;g__Coprococcus;s__ |
| OTU_30 | 89.64393487 | 1.57E-18 | 1.82E-15 | 5.47E-15 | 9.185185185 | 2.259259259 | 160.8888889 | 6.555555556 | 26.74074074 | k__Bacteria;p__Firmicutes;c__Clostridia;o__Clostridiales;f__Lachnospiraceae;g__Blautia;s__ |
| OTU_68 | 87.59876451 | 4.26E-18 | 3.72E-15 | 1.49E-14 | 2.851851852 | 1.925925926 | 66.18518519 | 6.074074074 | 11.11111111 | k__Bacteria;p__Firmicutes;c__Clostridia;o__Clostridiales;f__Lachnospiraceae;g__[Ruminococcus];s__ |
| OTU_50 | 86.41387447 | 7.60E-18 | 5.31E-15 | 2.65E-14 | 1.407407407 | 1.481481481 | 58.14814815 | 3.851851852 | 9.185185185 | k__Bacteria;p__Firmicutes;c__Erysipelotrichi;o__Erysipelotrichales;f__Erysipelotrichaceae;g__;s__ |
| OTU_5342 | 83.75531579 | 2.79E-17 | 1.40E-14 | 9.72E-14 | 0.185185185 | 0.37037037 | 8.37037037 | 0.592592593 | 1.925925926 | k__Bacteria;p__Firmicutes;c__Clostridia;o__Clostridiales;f__Lachnospiraceae |
| OTU_99 | 83.74271575 | 2.80E-17 | 1.40E-14 | 9.78E-14 | 1.111111111 | 0.296296296 | 24.62962963 | 1.555555556 | 3.814814815 | k__Bacteria;p__Firmicutes;c__Clostridia;o__Clostridiales;f__Lachnospiraceae;g__Dorea;s__formicigenerans |
| OTU_5139 | 82.60158972 | 4.89E-17 | 2.13E-14 | 1.71E-13 | 6.481481481 | 8.888888889 | 111.0740741 | 33.22222222 | 27.66666667 | k__Bacteria;p__Firmicutes;c__Clostridia;o__Clostridiales;f__Lachnospiraceae;g__Roseburia |
| OTU_64 | 79.92834926 | 1.80E-16 | 6.99E-14 | 6.30E-13 | 2.185185185 | 1.666666667 | 50.07407407 | 1.740740741 | 9.481481481 | k__Bacteria;p__Firmicutes;c__Clostridia;o__Clostridiales;f__Lachnospiraceae;g__Dorea;s__ |
| OTU_51 | 79.12128705 | 2.67E-16 | 9.33E-14 | 9.33E-13 | 2.666666667 | 3.444444444 | 54.96296296 | 4.074074074 | 13.37037037 | k__Bacteria;p__Firmicutes;c__Clostridia;o__Clostridiales;f__Lachnospiraceae;g__Blautia;s__obeum |
| OTU_132 | 78.42895586 | 3.75E-16 | 1.19E-13 | 1.31E-12 | 1.37037037 | 0.185185185 | 11 | 0.222222222 | 2.888888889 | k__Bacteria;p__Firmicutes;c__Clostridia;o__Clostridiales;f__Lachnospiraceae |
| OTU_113 | 72.73691922 | 6.00E-15 | 1.70E-12 | 2.09E-11 | 0.666666667 | 0.518518519 | 13.18518519 | 3.222222222 | 3.074074074 | k__Bacteria;p__Firmicutes;c__Clostridia;o__Clostridiales;f__Lachnospiraceae;g__Coprococcus;s__catus |
| OTU_17 | 72.63085608 | 6.31E-15 | 1.70E-12 | 2.20E-11 | 9.814814815 | 1.148148148 | 662.2222222 | 11.92592593 | 69.18518519 | k__Bacteria;p__Firmicutes;c__Clostridia;o__Clostridiales;f__Lachnospiraceae;g__Coprococcus;s__ |
| OTU_1895 | 71.6346996 | 1.03E-14 | 2.56E-12 | 3.58E-11 | 2.074074074 | 2.62962963 | 36.33333333 | 8.888888889 | 9.962962963 | k__Bacteria;p__Firmicutes;c__Clostridia;o__Clostridiales;f__Lachnospiraceae |
| OTU_3339 | 71.29871515 | 1.21E-14 | 2.81E-12 | 4.21E-11 | 0 | 0.037037037 | 1.259259259 | 0.074074074 | 0.148148148 | k__Bacteria;p__Firmicutes;c__Clostridia;o__Clostridiales;f__Lachnospiraceae |
| OTU_5018 | 70.36867096 | 1.90E-14 | 4.14E-12 | 6.62E-11 | 0 | 0.037037037 | 2.444444444 | 0.074074074 | 0.481481481 | k__Bacteria;p__Firmicutes;c__Clostridia;o__Clostridiales;f__Lachnospiraceae;g__Blautia;s__ |
| OTU_222 | 67.02240404 | 9.64E-14 | 1.98E-11 | 3.37E-10 | 0.555555556 | 1.259259259 | 13.33333333 | 4.296296296 | 3.333333333 | k__Bacteria;p__Firmicutes;c__Clostridia;o__Clostridiales;f__Lachnospiraceae;g__Roseburia |
| OTU_3978 | 64.39485433 | 3.45E-13 | 6.69E-11 | 1.20E-09 | 0 | 0 | 1.074074074 | 0 | 0.111111111 | k__Bacteria;p__Firmicutes;c__Clostridia;o__Clostridiales;f__Lachnospiraceae;g__Coprococcus;s__ |
| OTU_658 | 61.89167195 | 1.16E-12 | 2.13E-10 | 4.05E-09 | 0 | 0 | 1.296296296 | 0.037037037 | 0.185185185 | k__Bacteria;p__Firmicutes;c__Clostridia;o__Clostridiales;f__Lachnospiraceae;g__Coprococcus;s__ |
| OTU_526 | 61.64362239 | 1.31E-12 | 2.28E-10 | 4.57E-09 | 0.148148148 | 0.148148148 | 4.074074074 | 0.296296296 | 1 | k__Bacteria;p__Firmicutes;c__Clostridia;o__Clostridiales;f__Lachnospiraceae |
| OTU_4901 | 60.71025559 | 2.06E-12 | 3.42E-10 | 7.18E-09 | 0.222222222 | 0.037037037 | 2.592592593 | 0.185185185 | 0.518518519 | k__Bacteria;p__Firmicutes;c__Clostridia;o__Clostridiales;f__Lachnospiraceae;g__Coprococcus;s__ |
| OTU_61 | 59.28255444 | 4.10E-12 | 6.51E-10 | 1.43E-08 | 3.481481481 | 10.25925926 | 35.59259259 | 6.592592593 | 8.703703704 | k__Bacteria;p__Firmicutes;c__Clostridia;o__Clostridiales;f__Lachnospiraceae;g__Anaerostipes;s__ |
| OTU_2770 | 58.82023324 | 5.13E-12 | 7.60E-10 | 1.79E-08 | 0.148148148 | 0.481481481 | 5.222222222 | 0.925925926 | 0.666666667 | k__Bacteria;p__Firmicutes;c__Clostridia;o__Clostridiales;f__Lachnospiraceae |
| OTU_107 | 58.78204203 | 5.23E-12 | 7.60E-10 | 1.82E-08 | 8.222222222 | 7.333333333 | 4.62962963 | 2.037037037 | 1.259259259 | k__Bacteria;p__Firmicutes;c__Clostridia;o__Clostridiales;f__Ruminococcaceae;g__Oscillospira;s__ |
| OTU_5374 | 58.19252159 | 6.95E-12 | 9.71E-10 | 2.43E-08 | 0 | 0 | 1.888888889 | 0.037037037 | 0.481481481 | k__Bacteria;p__Firmicutes;c__Clostridia;o__Clostridiales |
| OTU_3879 | 58.09645889 | 7.28E-12 | 9.77E-10 | 2.54E-08 | 0.074074074 | 0.555555556 | 5.148148148 | 0.962962963 | 1.037037037 | k__Bacteria;p__Firmicutes;c__Clostridia;o__Clostridiales;f__Lachnospiraceae;g__Roseburia |
| OTU_44 | 58.0194212 | 7.56E-12 | 9.77E-10 | 2.64E-08 | 2.925925926 | 1.851851852 | 89 | 4.296296296 | 5.703703704 | k__Bacteria;p__Firmicutes;c__Clostridia;o__Clostridiales;f__Clostridiaceae;g__SMB53;s__ |
| OTU_5044 | 57.8769639 | 8.10E-12 | 1.01E-09 | 2.83E-08 | 1.222222222 | 0.666666667 | 24.22222222 | 2.925925926 | 5.592592593 | k__Bacteria;p__Firmicutes;c__Clostridia;o__Clostridiales;f__Lachnospiraceae;g__Blautia;s__ |
| OTU_3446 | 57.73185119 | 8.69E-12 | 1.02E-09 | 3.03E-08 | 0.111111111 | 0.222222222 | 2.703703704 | 0.740740741 | 0.37037037 | k__Bacteria;p__Firmicutes;c__Clostridia;o__Clostridiales;f__Lachnospiraceae |
| OTU_4004 | 57.71651721 | 8.75E-12 | 1.02E-09 | 3.05E-08 | 0 | 0 | 1.62962963 | 0.037037037 | 0.259259259 | k__Bacteria;p__Firmicutes;c__Clostridia;o__Clostridiales;f__Lachnospiraceae;g__Coprococcus;s__ |
| OTU_2368 | 56.69603132 | 1.43E-11 | 1.61E-09 | 5.00E-08 | 0 | 0 | 3.925925926 | 0.111111111 | 1.148148148 | k__Bacteria;p__Firmicutes;c__Clostridia;o__Clostridiales;f__Lachnospiraceae;g__Coprococcus;s__ |
| OTU_3479 | 55.01113537 | 3.23E-11 | 3.52E-09 | 1.13E-07 | 0.037037037 | 0 | 4.259259259 | 0.111111111 | 0.444444444 | k__Bacteria;p__Firmicutes;c__Clostridia;o__Clostridiales;f__Lachnospiraceae |
| OTU_263 | 54.22765248 | 4.72E-11 | 4.99E-09 | 1.65E-07 | 0.037037037 | 0 | 1.037037037 | 0.037037037 | 0.074074074 | k__Bacteria;p__Firmicutes;c__Erysipelotrichi;o__Erysipelotrichales;f__Erysipelotrichaceae;g__[Eubacterium];s__dolichum |
| OTU_3249 | 52.90577441 | 8.92E-11 | 9.15E-09 | 3.11E-07 | 0.074074074 | 0.111111111 | 1.925925926 | 0.148148148 | 0.592592593 | k__Bacteria;p__Firmicutes;c__Clostridia;o__Clostridiales;f__Lachnospiraceae |
| OTU_372 | 52.21031362 | 1.25E-10 | 1.24E-08 | 4.35E-07 | 0.222222222 | 0 | 2.925925926 | 0.333333333 | 0.740740741 | k__Bacteria;p__Firmicutes;c__Clostridia;o__Clostridiales;f__Lachnospiraceae;g__Blautia;s__ |
| OTU_2526 | 52.06220583 | 1.34E-10 | 1.30E-08 | 4.67E-07 | 0.592592593 | 0.777777778 | 10.37037037 | 3.592592593 | 1.555555556 | k__Bacteria;p__Firmicutes;c__Clostridia;o__Clostridiales;f__Lachnospiraceae |
| OTU_2261 | 51.99700658 | 1.38E-10 | 1.30E-08 | 4.82E-07 | 0 | 0 | 0.740740741 | 0 | 0.037037037 | k__Bacteria;p__Firmicutes;c__Clostridia;o__Clostridiales;f__Lachnospiraceae;g__Blautia;s__ |
| OTU_127 | 51.77373034 | 1.54E-10 | 1.41E-08 | 5.37E-07 | 0.666666667 | 1 | 7.777777778 | 0.851851852 | 2.111111111 | k__Bacteria;p__Firmicutes;c__Bacilli;o__Lactobacillales;f__Streptococcaceae;g__Streptococcus;s__ |
| OTU_3795 | 51.44113679 | 1.81E-10 | 1.59E-08 | 6.30E-07 | 0.037037037 | 0 | 1.407407407 | 0 | 0.111111111 | k__Bacteria;p__Firmicutes;c__Clostridia;o__Clostridiales;f__Lachnospiraceae;g__;s__ |
| OTU_1432 | 51.41826717 | 1.83E-10 | 1.59E-08 | 6.37E-07 | 0.074074074 | 0.037037037 | 0.814814815 | 0.037037037 | 0.074074074 | k__Bacteria;p__Firmicutes;c__Clostridia;o__Clostridiales;f__Lachnospiraceae |
| OTU_94 | 51.27746 | 1.95E-10 | 1.65E-08 | 6.82E-07 | 4.481481481 | 10.11111111 | 10.74074074 | 1.296296296 | 3.555555556 | k__Bacteria;p__Firmicutes;c__Clostridia;o__Clostridiales;f__Lachnospiraceae |
| OTU_515 | 51.24459649 | 1.98E-10 | 1.65E-08 | 6.93E-07 | 0.148148148 | 0.037037037 | 2.592592593 | 0 | 0.444444444 | k__Bacteria;p__Firmicutes;c__Clostridia;o__Clostridiales;f__Lachnospiraceae;g__Blautia;s__ |
| OTU_363 | 51.09701765 | 2.13E-10 | 1.73E-08 | 7.44E-07 | 0.592592593 | 0.518518519 | 4.259259259 | 1.851851852 | 1.703703704 | k__Bacteria;p__Firmicutes;c__Clostridia;o__Clostridiales;f__Lachnospiraceae |
| OTU_722 | 51.02450376 | 2.21E-10 | 1.75E-08 | 7.70E-07 | 0.148148148 | 0 | 9.296296296 | 0.222222222 | 0.851851852 | k__Bacteria;p__Firmicutes;c__Clostridia;o__Clostridiales;f__Lachnospiraceae;g__Coprococcus;s__ |
| OTU_4142 | 50.67725861 | 2.61E-10 | 2.02E-08 | 9.10E-07 | 0 | 0 | 2.296296296 | 0.037037037 | 0.37037037 | k__Bacteria;p__Firmicutes;c__Clostridia;o__Clostridiales;f__Lachnospiraceae;g__Coprococcus;s__ |
| OTU_5232 | 47.90233107 | 9.89E-10 | 7.50E-08 | 3.45E-06 | 0.037037037 | 0 | 1.148148148 | 0 | 0.037037037 | k__Bacteria;p__Firmicutes;c__Clostridia;o__Clostridiales |
| OTU_5073 | 46.83107942 | 1.65E-09 | 1.20E-07 | 5.77E-06 | 1.777777778 | 1.925925926 | 17.59259259 | 3.185185185 | 6.962962963 | k__Bacteria;p__Firmicutes;c__Clostridia;o__Clostridiales;f__Lachnospiraceae |
| OTU_5180 | 46.82812937 | 1.66E-09 | 1.20E-07 | 5.78E-06 | 0.444444444 | 0.851851852 | 5.111111111 | 1.37037037 | 1.111111111 | k__Bacteria;p__Firmicutes;c__Clostridia;o__Clostridiales;f__Lachnospiraceae;g__Roseburia |
| OTU_3913 | 46.22012798 | 2.22E-09 | 1.58E-07 | 7.73E-06 | 0 | 0 | 1.888888889 | 0.037037037 | 0.111111111 | k__Bacteria;p__Firmicutes;c__Clostridia;o__Clostridiales;f__Lachnospiraceae |
| OTU_3681 | 45.60229947 | 2.98E-09 | 2.08E-07 | 1.04E-05 | 3.407407407 | 2.925925926 | 8.37037037 | 2.259259259 | 2.444444444 | k__Bacteria;p__Firmicutes;c__Clostridia;o__Clostridiales;f__Lachnospiraceae |
| OTU_4336 | 45.16899283 | 3.67E-09 | 2.51E-07 | 1.28E-05 | 0.185185185 | 0.444444444 | 5.111111111 | 1.074074074 | 1.518518519 | k__Bacteria;p__Firmicutes;c__Clostridia;o__Clostridiales;f__Lachnospiraceae;g__Roseburia |
| OTU_1740 | 44.87207454 | 4.23E-09 | 2.84E-07 | 1.48E-05 | 4.333333333 | 5.296296296 | 0.518518519 | 2.148148148 | 2.703703704 | k__Bacteria;p__Bacteroidetes;c__Bacteroidia;o__Bacteroidales;f__Porphyromonadaceae;g__Parabacteroides;s__distasonis |
| OTU_5040 | 43.65540894 | 7.57E-09 | 4.98E-07 | 2.64E-05 | 0.037037037 | 0.037037037 | 1.074074074 | 0.037037037 | 0.259259259 | k__Bacteria;p__Firmicutes;c__Clostridia;o__Clostridiales;f__Lachnospiraceae |
| OTU_3213 | 43.20309663 | 9.39E-09 | 6.07E-07 | 3.28E-05 | 0 | 0 | 0.555555556 | 0 | 0.074074074 | k__Bacteria;p__Firmicutes;c__Clostridia;o__Clostridiales;f__Lachnospiraceae;g__Coprococcus;s__ |
| OTU_3065 | 42.66763958 | 1.21E-08 | 7.70E-07 | 4.23E-05 | 0 | 0 | 1.037037037 | 0 | 0.037037037 | k__Bacteria;p__Firmicutes;c__Clostridia;o__Clostridiales;f__Lachnospiraceae |
| OTU_4675 | 42.41108582 | 1.37E-08 | 8.54E-07 | 4.78E-05 | 1 | 0.333333333 | 3.407407407 | 0.444444444 | 0.814814815 | k__Bacteria;p__Firmicutes;c__Clostridia;o__Clostridiales;f__Ruminococcaceae;g__Faecalibacterium |
| OTU_67 | 41.6981828 | 1.93E-08 | 1.18E-06 | 6.72E-05 | 0.851851852 | 0.148148148 | 53.77777778 | 1.37037037 | 10.11111111 | k__Bacteria;p__Firmicutes;c__Clostridia;o__Clostridiales |
| OTU_130 | 41.65159079 | 1.97E-08 | 1.19E-06 | 6.87E-05 | 0.074074074 | 0.222222222 | 6.740740741 | 0.148148148 | 0.111111111 | k__Bacteria;p__Firmicutes;c__Bacilli;o__Lactobacillales;f__Streptococcaceae;g__Streptococcus |
| OTU_1499 | 40.46623367 | 3.47E-08 | 2.05E-06 | 0.000120978 | 0 | 0.074074074 | 1.074074074 | 0.037037037 | 0.259259259 | k__Bacteria;p__Firmicutes;c__Clostridia;o__Clostridiales;f__Lachnospiraceae |
| OTU_4047 | 40.02627622 | 4.27E-08 | 2.49E-06 | 0.000149184 | 0 | 0 | 2.074074074 | 0.074074074 | 0.259259259 | k__Bacteria;p__Firmicutes;c__Clostridia;o__Clostridiales;f__Lachnospiraceae |
| OTU_809 | 39.72541769 | 4.93E-08 | 2.82E-06 | 0.00017216 | 0.037037037 | 0 | 0.62962963 | 0.037037037 | 0.074074074 | k__Bacteria;p__Firmicutes;c__Clostridia;o__Clostridiales;f__Lachnospiraceae;g__Coprococcus;s__ |
| OTU_523 | 39.52443352 | 5.43E-08 | 3.06E-06 | 0.000189443 | 0.333333333 | 0.925925926 | 2.814814815 | 0.333333333 | 1.148148148 | k__Bacteria;p__Firmicutes;c__Clostridia;o__Clostridiales;f__Lachnospiraceae;g__Roseburia |
| OTU_3053 | 39.44799211 | 5.63E-08 | 3.12E-06 | 0.000196461 | 0.037037037 | 0 | 0.666666667 | 0 | 0.074074074 | k__Bacteria;p__Firmicutes;c__Clostridia;o__Clostridiales;f__Lachnospiraceae;g__Coprococcus;s__ |
| OTU_95 | 39.21400166 | 6.29E-08 | 3.43E-06 | 0.000219598 | 3.185185185 | 6.518518519 | 13.7037037 | 3.111111111 | 3.222222222 | k__Bacteria;p__Firmicutes;c__Clostridia;o__Clostridiales;f__Ruminococcaceae;g__Ruminococcus;s__ |
| OTU_73 | 39.02891893 | 6.87E-08 | 3.69E-06 | 0.000239808 | 10.25925926 | 18.22222222 | 16.25925926 | 4.259259259 | 5.740740741 | k__Bacteria;p__Firmicutes;c__Clostridia;o__Clostridiales;f__Lachnospiraceae;g__;s__ |
| OTU_2260 | 38.32091832 | 9.62E-08 | 5.09E-06 | 0.000335772 | 0.111111111 | 0 | 1.592592593 | 0.185185185 | 0.592592593 | k__Bacteria;p__Firmicutes;c__Clostridia;o__Clostridiales;f__Lachnospiraceae;g__Blautia |
| OTU_36 | 37.94293564 | 1.15E-07 | 5.92E-06 | 0.00040182 | 47.03703704 | 37.51851852 | 14.77777778 | 22.44444444 | 14.51851852 | k__Bacteria;p__Proteobacteria;c__Deltaproteobacteria;o__Desulfovibrionales;f__Desulfovibrionaceae;g__Bilophila;s__ |
| OTU_2497 | 37.93751362 | 1.15E-07 | 5.92E-06 | 0.000402856 | 5.481481481 | 7 | 47.37037037 | 9.037037037 | 17.40740741 | k__Bacteria;p__Firmicutes;c__Clostridia;o__Clostridiales;f__Lachnospiraceae;g__Roseburia;s__faecis |
| OTU_4486 | 37.78990916 | 1.24E-07 | 6.23E-06 | 0.00043211 | 100.1481481 | 47.7037037 | 200.4074074 | 46.59259259 | 71.11111111 | k__Bacteria;p__Firmicutes;c__Clostridia;o__Clostridiales;f__Ruminococcaceae;g__Faecalibacterium |
| OTU_2600 | 37.77021448 | 1.25E-07 | 6.23E-06 | 0.00043617 | 0 | 0 | 1.148148148 | 0 | 0.074074074 | k__Bacteria;p__Firmicutes;c__Bacilli;o__Lactobacillales;f__Lactobacillaceae |
| OTU_4480 | 37.52370159 | 1.40E-07 | 6.91E-06 | 0.000490326 | 0.407407407 | 0.296296296 | 2.740740741 | 1.037037037 | 0.814814815 | k__Bacteria;p__Firmicutes;c__Clostridia;o__Clostridiales;f__Lachnospiraceae;g__Roseburia |
| OTU_4681 | 37.29045314 | 1.57E-07 | 7.61E-06 | 0.000547726 | 4.518518519 | 7.296296296 | 6.222222222 | 0.481481481 | 2.333333333 | k__Bacteria;p__Firmicutes;c__Clostridia;o__Clostridiales;f__Lachnospiraceae;g__;s__ |
| OTU_923 | 37.22102111 | 1.62E-07 | 7.75E-06 | 0.000566073 | 0.037037037 | 0.111111111 | 1 | 0.037037037 | 0.333333333 | k__Bacteria;p__Firmicutes;c__Clostridia;o__Clostridiales;f__Lachnospiraceae;g__Blautia;s__ |
| OTU_104 | 36.76098888 | 2.02E-07 | 9.52E-06 | 0.000704114 | 3.592592593 | 0.296296296 | 7.888888889 | 0.814814815 | 1.444444444 | k__Bacteria;p__Firmicutes;c__Clostridia;o__Clostridiales;f__Ruminococcaceae;g__Oscillospira;s__ |
| OTU_3800 | 36.7258862 | 2.05E-07 | 9.55E-06 | 0.000715932 | 1.555555556 | 2.851851852 | 13.7037037 | 2.185185185 | 5.37037037 | k__Bacteria;p__Firmicutes;c__Clostridia;o__Clostridiales;f__Lachnospiraceae;g__Roseburia |
| OTU_243 | 35.95404162 | 2.96E-07 | 1.36E-05 | 0.001032126 | 0.037037037 | 0.037037037 | 0.777777778 | 0.037037037 | 0.259259259 | k__Bacteria;p__Firmicutes;c__Clostridia;o__Clostridiales;f__Lachnospiraceae;g__Blautia;s__producta |
| OTU_58 | 35.73097364 | 3.29E-07 | 1.48E-05 | 0.001147126 | 3.333333333 | 2.296296296 | 50.51851852 | 5.703703704 | 26.25925926 | k__Bacteria;p__Actinobacteria;c__Actinobacteria;o__Bifidobacteriales;f__Bifidobacteriaceae;g__Bifidobacterium;s__adolescentis |
| OTU_5281 | 35.71268594 | 3.32E-07 | 1.48E-05 | 0.001157102 | 0.111111111 | 0.296296296 | 2.185185185 | 0.296296296 | 0.814814815 | k__Bacteria;p__Firmicutes;c__Clostridia;o__Clostridiales;f__Lachnospiraceae |
| OTU_547 | 35.31138347 | 4.01E-07 | 1.77E-05 | 0.00139916 | 0.481481481 | 0.296296296 | 3.037037037 | 0.814814815 | 1.37037037 | k__Bacteria;p__Firmicutes;c__Clostridia;o__Clostridiales;f__Lachnospiraceae;g__Roseburia;s__faecis |
| OTU_941 | 35.29023122 | 4.05E-07 | 1.77E-05 | 0.001413234 | 1.074074074 | 0.703703704 | 5.62962963 | 0.777777778 | 1.222222222 | k__Bacteria;p__Firmicutes;c__Clostridia;o__Clostridiales;f__Ruminococcaceae;g__Faecalibacterium |
| OTU_5286 | 35.17071715 | 4.28E-07 | 1.85E-05 | 0.001495451 | 0.074074074 | 0.074074074 | 1.962962963 | 0.111111111 | 0.074074074 | k__Bacteria;p__Firmicutes;c__Clostridia;o__Clostridiales;f__Lachnospiraceae |
| OTU_3601 | 34.90622335 | 4.86E-07 | 2.07E-05 | 0.001694747 | 0.037037037 | 0 | 0.555555556 | 0 | 0.074074074 | k__Bacteria;p__Firmicutes;c__Clostridia;o__Clostridiales;f__Lachnospiraceae;g__Coprococcus;s__ |
| OTU_1161 | 33.56288534 | 9.16E-07 | 3.85E-05 | 0.003196719 | 3.777777778 | 1.407407407 | 7.814814815 | 2.333333333 | 2.703703704 | k__Bacteria;p__Firmicutes;c__Clostridia;o__Clostridiales;f__Ruminococcaceae;g__Faecalibacterium;s__prausnitzii |
| OTU_3646 | 33.49393715 | 9.46E-07 | 3.93E-05 | 0.00330243 | 0 | 0 | 0.814814815 | 0 | 0.037037037 | k__Bacteria;p__Firmicutes;c__Clostridia;o__Clostridiales;f__Lachnospiraceae;g__Coprococcus;s__ |
| OTU_2491 | 33.38156566 | 9.98E-07 | 4.10E-05 | 0.003482231 | 0 | 0 | 0.444444444 | 0 | 0.037037037 | k__Bacteria;p__Firmicutes;c__Clostridia;o__Clostridiales;f__Lachnospiraceae;g__Blautia;s__ |
| OTU_1799 | 33.03688577 | 1.17E-06 | 4.76E-05 | 0.004096871 | 0.037037037 | 0 | 0.777777778 | 0 | 0.111111111 | k__Bacteria;p__Firmicutes;c__Clostridia;o__Clostridiales;f__Lachnospiraceae;g__Coprococcus;s__ |
| OTU_3086 | 33.01043255 | 1.19E-06 | 4.77E-05 | 0.004148285 | 4.296296296 | 0.740740741 | 3.925925926 | 0.444444444 | 4.333333333 | k__Bacteria;p__Firmicutes;c__Clostridia;o__Clostridiales;f__Ruminococcaceae |
| OTU_1639 | 32.73805413 | 1.35E-06 | 5.36E-05 | 0.004716532 | 1.518518519 | 0.703703704 | 3 | 0.518518519 | 1.592592593 | k__Bacteria;p__Firmicutes;c__Clostridia;o__Clostridiales;f__Ruminococcaceae;g__Faecalibacterium |
| OTU_3398 | 32.64811599 | 1.41E-06 | 5.53E-05 | 0.004920698 | 4.37037037 | 6.407407407 | 1.518518519 | 6.925925926 | 3.592592593 | k__Bacteria;p__Bacteroidetes;c__Bacteroidia;o__Bacteroidales;f__Bacteroidaceae;g__Bacteroides;s__ |
| OTU_1531 | 32.45193567 | 1.55E-06 | 6.00E-05 | 0.005397104 | 0.407407407 | 0.185185185 | 4.407407407 | 0.222222222 | 0.888888889 | k__Bacteria;p__Firmicutes;c__Clostridia;o__Clostridiales;f__Lachnospiraceae |
| OTU_2797 | 32.34426222 | 1.63E-06 | 6.24E-05 | 0.005677829 | 0 | 0.037037037 | 0.592592593 | 0.074074074 | 0.185185185 | k__Bacteria;p__Firmicutes;c__Clostridia;o__Clostridiales;f__Lachnospiraceae;g__Blautia;s__ |
| OTU_1267 | 32.08162295 | 1.84E-06 | 6.98E-05 | 0.006425099 | 0.074074074 | 0.222222222 | 3.222222222 | 0.222222222 | 0.333333333 | k__Bacteria;p__Firmicutes;c__Bacilli;o__Lactobacillales;f__Streptococcaceae;g__Streptococcus;s__ |
| OTU_79 | 31.92697482 | 1.98E-06 | 7.43E-05 | 0.006910128 | 6.62962963 | 12.88888889 | 19.66666667 | 1.481481481 | 5.074074074 | k__Bacteria;p__Firmicutes;c__Clostridia;o__Clostridiales;f__Lachnospiraceae;g__;s__ |
| OTU_344 | 31.71132414 | 2.19E-06 | 8.14E-05 | 0.007647944 | 6.111111111 | 2.777777778 | 12.85185185 | 2.962962963 | 4.962962963 | k__Bacteria;p__Firmicutes;c__Clostridia;o__Clostridiales;f__Ruminococcaceae;g__Faecalibacterium;s__prausnitzii |
| OTU_239 | 31.47911148 | 2.44E-06 | 8.98E-05 | 0.008530356 | 0.037037037 | 0 | 0.037037037 | 0.888888889 | 0.074074074 | k__Bacteria;p__Firmicutes;c__Clostridia;o__Clostridiales;f__;g__;s__ |
| OTU_198 | 31.10275196 | 2.92E-06 | 0.000106 | 0.01018082 | 0.888888889 | 1.222222222 | 0.740740741 | 0.259259259 | 0.037037037 | k__Bacteria;p__Firmicutes;c__Clostridia;o__Clostridiales |
| OTU_2254 | 30.78485754 | 3.39E-06 | 0.000122 | 0.011820119 | 0.074074074 | 0.037037037 | 1.740740741 | 0.333333333 | 0.62962963 | k__Bacteria;p__Firmicutes;c__Clostridia;o__Clostridiales;f__Lachnospiraceae;g__Blautia |
| OTU_134 | 30.15722777 | 4.55E-06 | 0.000162 | 0.015867746 | 3.814814815 | 4.037037037 | 1.555555556 | 1.296296296 | 0.259259259 | k__Bacteria;p__Firmicutes;c__Clostridia;o__Clostridiales;f__Ruminococcaceae;g__Oscillospira;s__ |
| OTU_4722 | 29.5549459 | 6.03E-06 | 0.000213 | 0.021042043 | 0.074074074 | 0.148148148 | 15.33333333 | 0.703703704 | 1.703703704 | k__Bacteria;p__Firmicutes;c__Clostridia;o__Clostridiales;f__Lachnospiraceae |
| OTU_1861 | 29.5265069 | 6.11E-06 | 0.000213 | 0.021324152 | 0 | 0 | 0.703703704 | 0.037037037 | 0.037037037 | k__Bacteria;p__Firmicutes;c__Clostridia;o__Clostridiales;f__Lachnospiraceae;g__Coprococcus;s__ |
| OTU_3491 | 29.30232558 | 6.79E-06 | 0.000232 | 0.023683882 | 0 | 0 | 0.296296296 | 0 | 0 | k__Bacteria;p__Firmicutes;c__Clostridia;o__Clostridiales;f__Lachnospiraceae;g__Blautia;s__ |
| OTU_3531 | 29.30200623 | 6.79E-06 | 0.000232 | 0.023687422 | 0 | 0 | 0.481481481 | 0.037037037 | 0.037037037 | k__Bacteria;p__Firmicutes;c__Clostridia;o__Clostridiales;f__Lachnospiraceae;g__Coprococcus;s__ |
| OTU_1 | 29.21231814 | 7.08E-06 | 0.00024 | 0.024702856 | 20.77777778 | 21.51851852 | 6.62962963 | 15.03703704 | 11.74074074 | k__Bacteria;p__Bacteroidetes;c__Bacteroidia;o__Bacteroidales;f__Bacteroidaceae;g__Bacteroides;s__ |
| OTU_4661 | 29.16240922 | 7.25E-06 | 0.000243 | 0.02528656 | 0 | 0 | 0.592592593 | 0 | 0.037037037 | k__Bacteria;p__Firmicutes;c__Clostridia;o__Clostridiales;f__Lachnospiraceae |
| OTU_2527 | 29.10389696 | 7.45E-06 | 0.000248 | 0.025988386 | 3.333333333 | 1.259259259 | 4.740740741 | 1.37037037 | 1.888888889 | k__Bacteria;p__Firmicutes;c__Clostridia;o__Clostridiales;f__Ruminococcaceae;g__Faecalibacterium;s__prausnitzii |
| OTU_4804 | 28.84165718 | 8.42E-06 | 0.000277 | 0.029379666 | 0.074074074 | 0 | 1.259259259 | 0.037037037 | 0.074074074 | k__Bacteria;p__Firmicutes;c__Clostridia;o__Clostridiales;f__Lachnospiraceae;g__[Ruminococcus];s__gnavus |
| OTU_310 | 28.73632095 | 8.84E-06 | 0.000288 | 0.030862742 | 0 | 0 | 2.222222222 | 0.148148148 | 0.111111111 | k__Bacteria;p__Firmicutes;c__Clostridia;o__Clostridiales;f__Lachnospiraceae |
| OTU_165 | 28.58318358 | 9.50E-06 | 0.000307 | 0.033152682 | 1.259259259 | 1.037037037 | 2.259259259 | 0.222222222 | 1.333333333 | k__Bacteria;p__Firmicutes;c__Clostridia;o__Clostridiales;f__[Mogibacteriaceae];g__;s__ |
| OTU_630 | 28.03961286 | 1.22E-05 | 0.000392 | 0.042733178 | 2.851851852 | 2.555555556 | 0.259259259 | 2.518518519 | 1.777777778 | k__Bacteria;p__Bacteroidetes;c__Bacteroidia;o__Bacteroidales;f__Bacteroidaceae;g__Bacteroides;s__ |
| OTU_1083 | 27.75330809 | 1.40E-05 | 0.000444 | 0.048840085 | 0.037037037 | 0.148148148 | 1.111111111 | 0.259259259 | 0.37037037 | k__Bacteria;p__Firmicutes;c__Clostridia;o__Clostridiales;f__Lachnospiraceae;g__Roseburia;s__ |
